# Supplementary material for: Decoding the dual recognition mechanism of the glucocorticoid receptor for DNA and RNA: sequence versus shape
Source: Sci Rep. 2023 Sep 26;13:16125. doi: 10.1038/s41598-023-43244-1 (PMC10522765; doi:10.1038/s41598-023-43244-1)
Supplement: Supplementary file 1 — Supplementary Information 1. [file 41598_2023_43244_MOESM1_ESM.pdf]

Decoding the Dual Recognition Mechanism of Glucocorticoid Receptor for DNA and RNA: Sequence vs. Shape

Hörberg Johanna & Reymer* Anna
Department of Chemistry and Molecular Biology, University of Gothenburg, 405 30 Göteborg, Sweden. * Email to: anna.reymer@gu.se

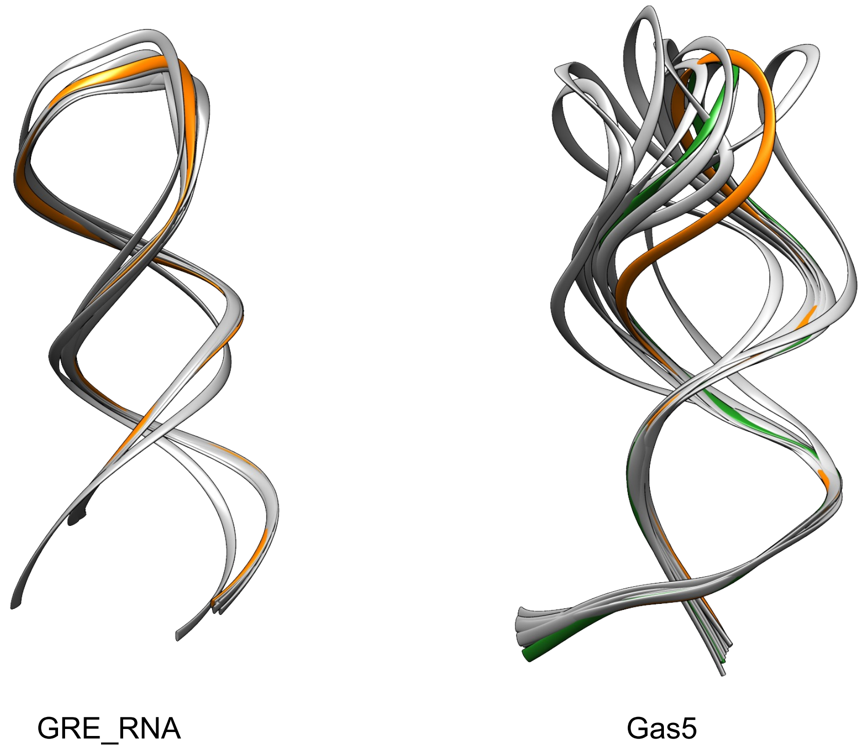

Figure S1: Derived models of GRE_RNA and Gas5_RNA. Models highlighted with orange (the highest ranked model) and green (the second highest ranked model) were used for the docking studies.

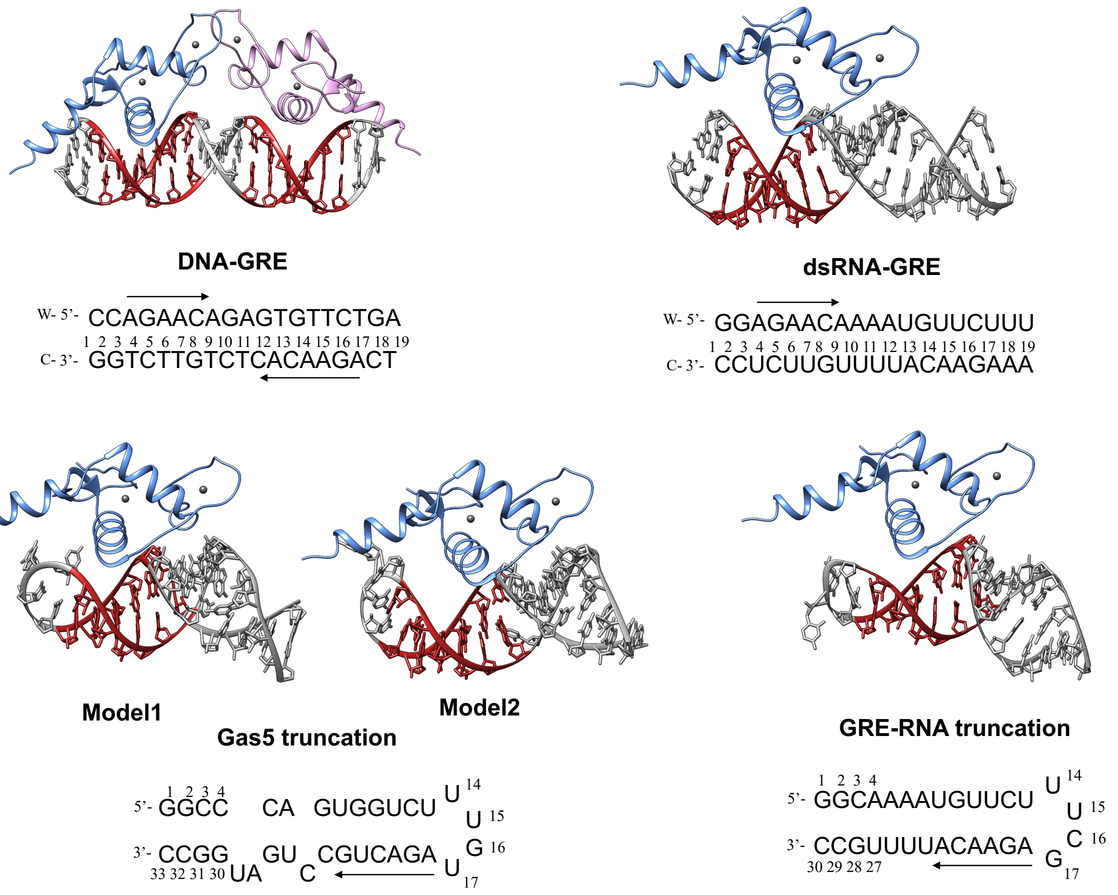

Figure S2: Prepared GR-DNA/RNA complexes.

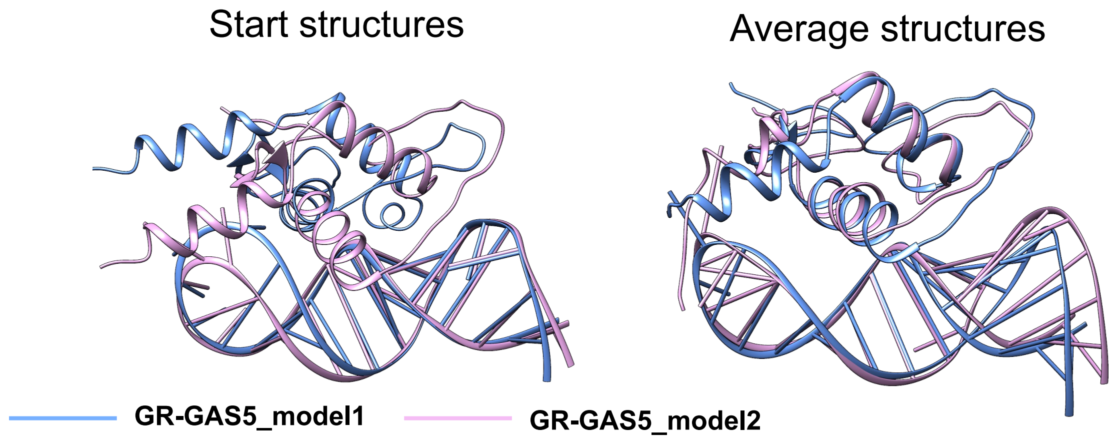

Figure S3: Comparison of GR-Gas5 model1 and model2 starting and average structures.

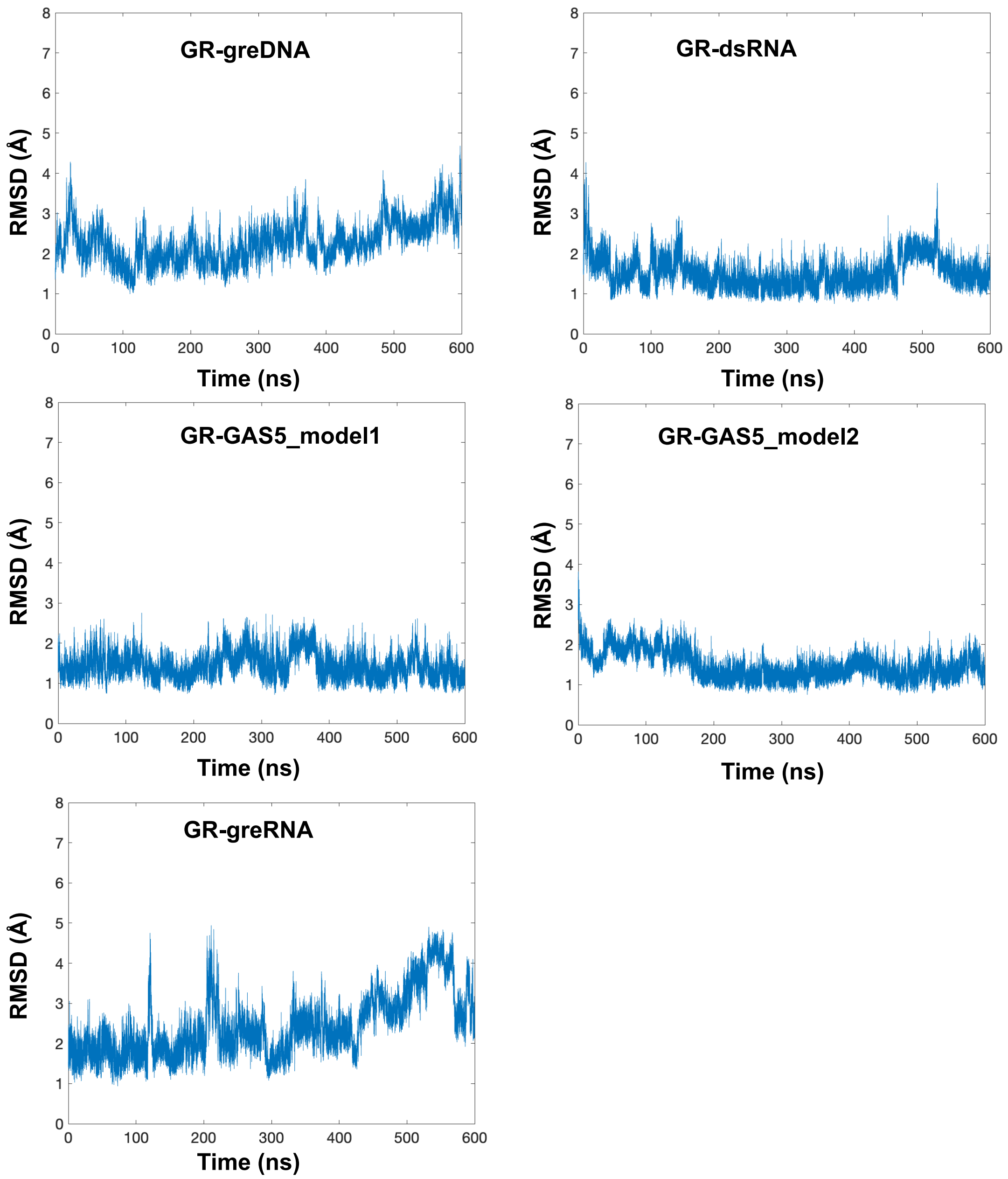

Figure S4: Fluctuations of protein heavy atoms RMSD relative to the average structures for the whole md trajectory.

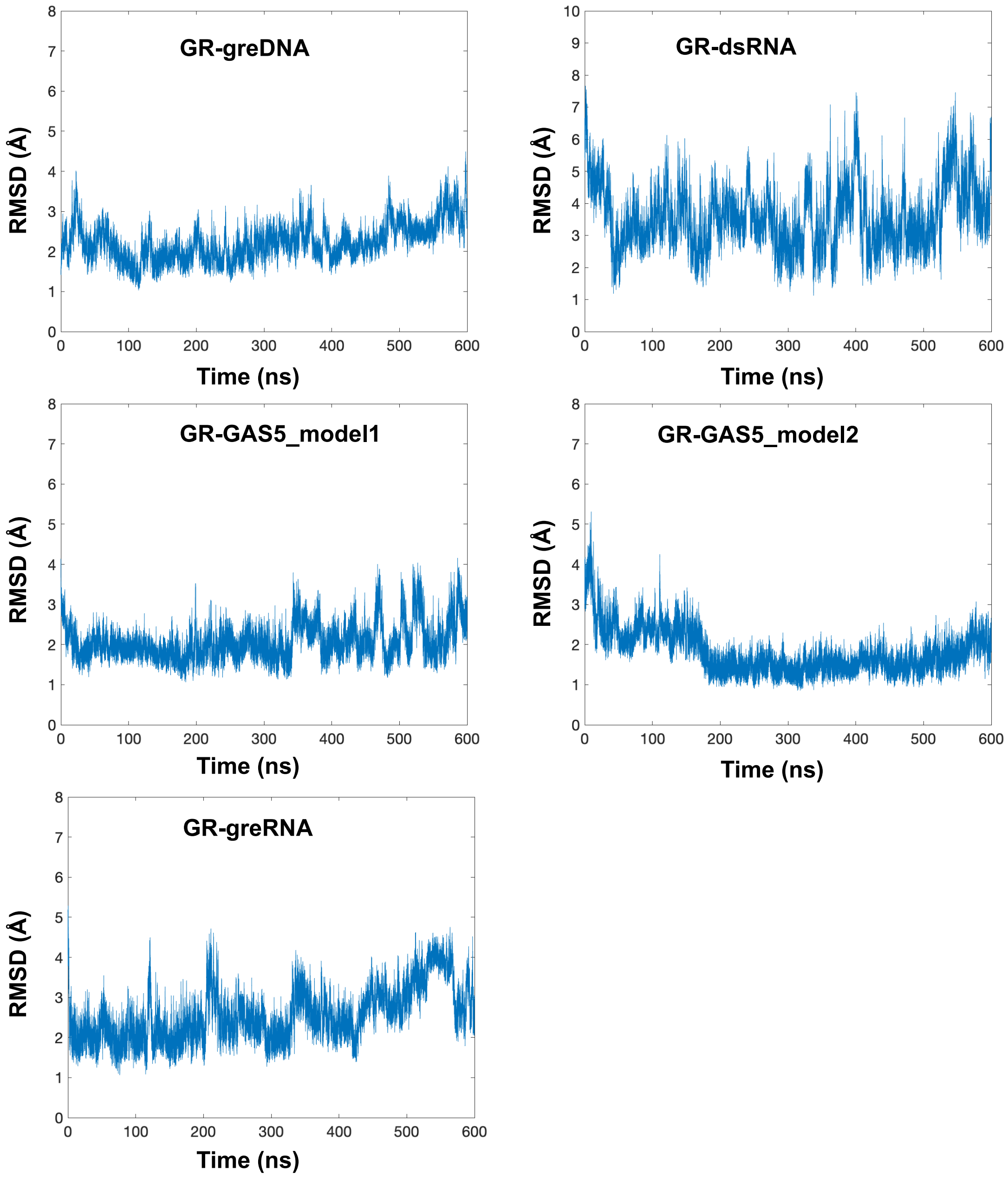

Figure S5: Fluctuations of protein-DNA/RNA complex heavy atoms RMSD relative to the average structures for the whole md trajectory.

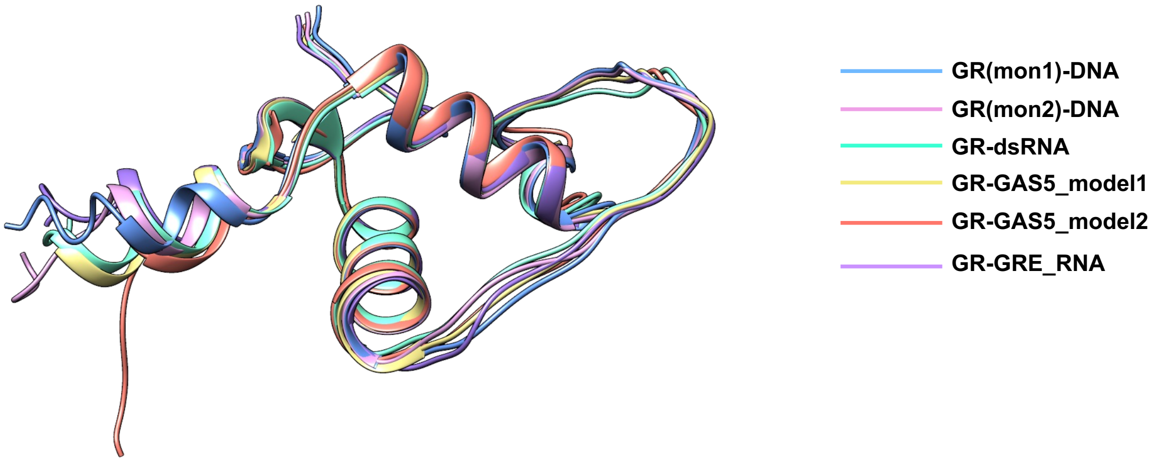

Figure S6: Comparison of GR from all average structures, illustrating that predominant conformational changes occur at helix4.

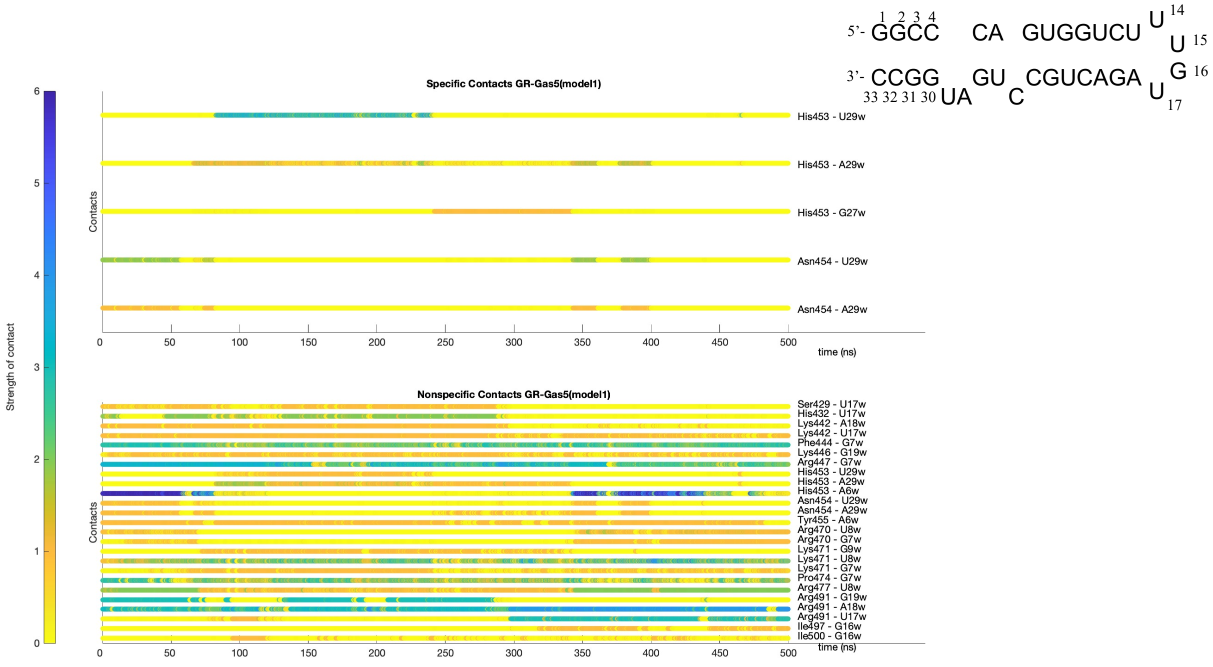

Figure S7: Dynamic contacts map for the specific and nonspecific GR-Gas5_RNA_model1 interactions.

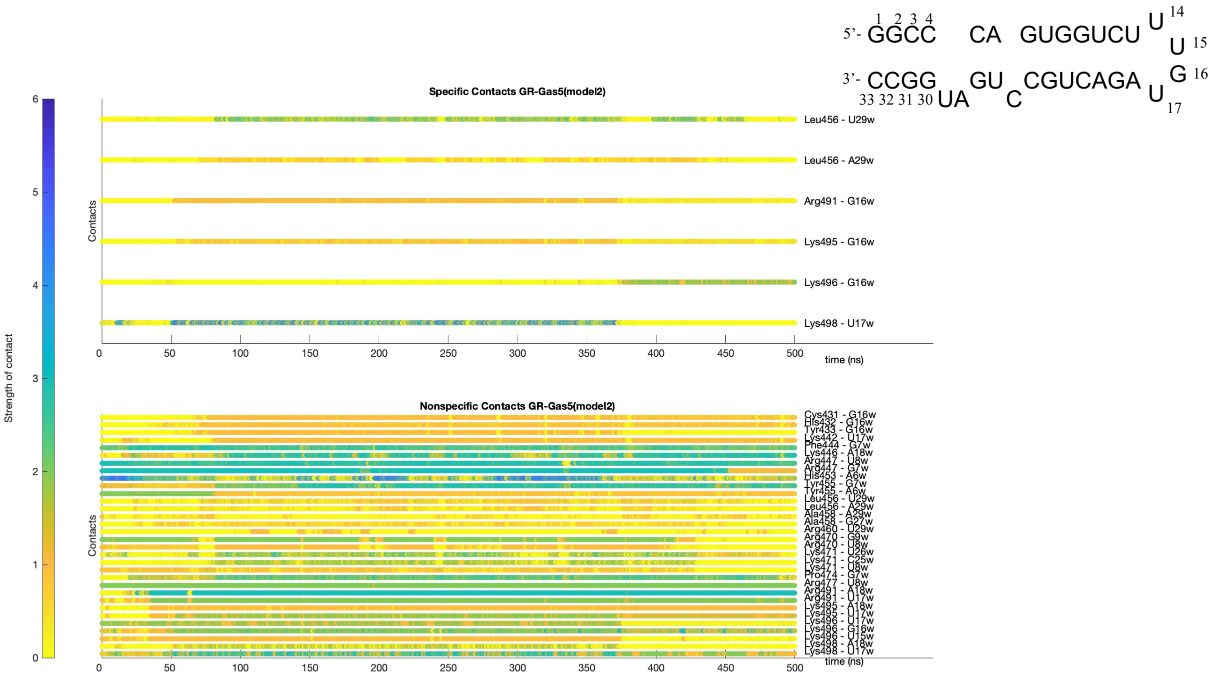

Figure S8: Dynamic contacts map for the specific and nonspecific GR-Gas5_RNA_model2 interactions.

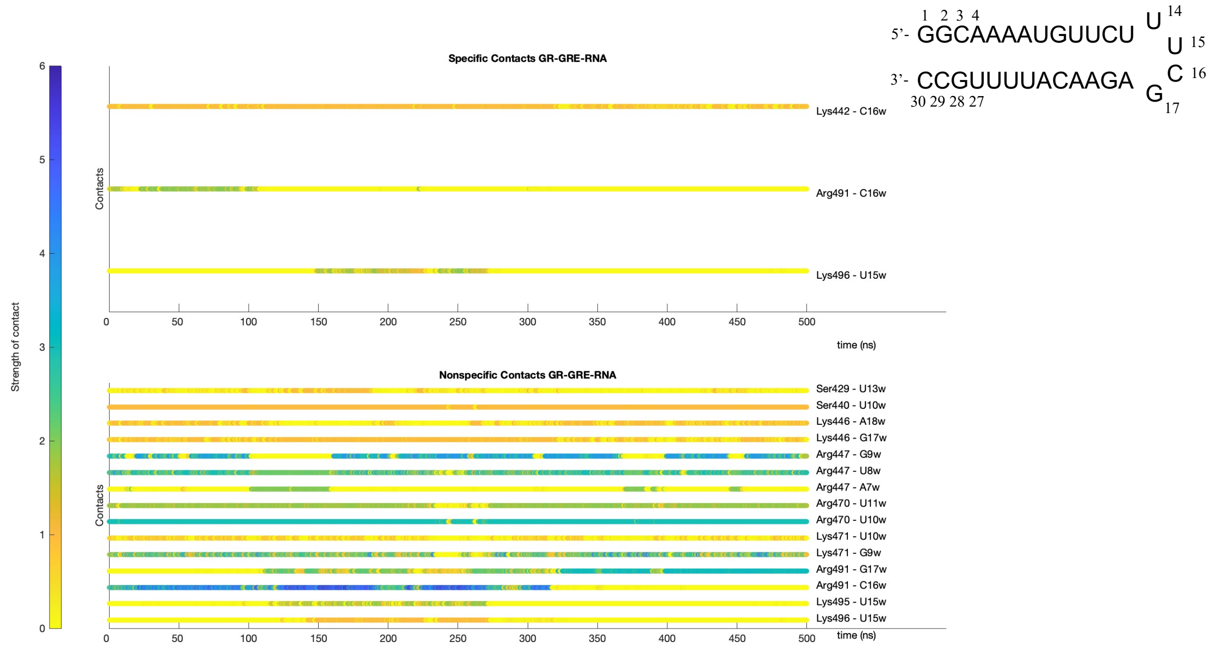

Figure S9: Dynamic contacts map for the specific and nonspecific GR-GRE_RNA interactions.

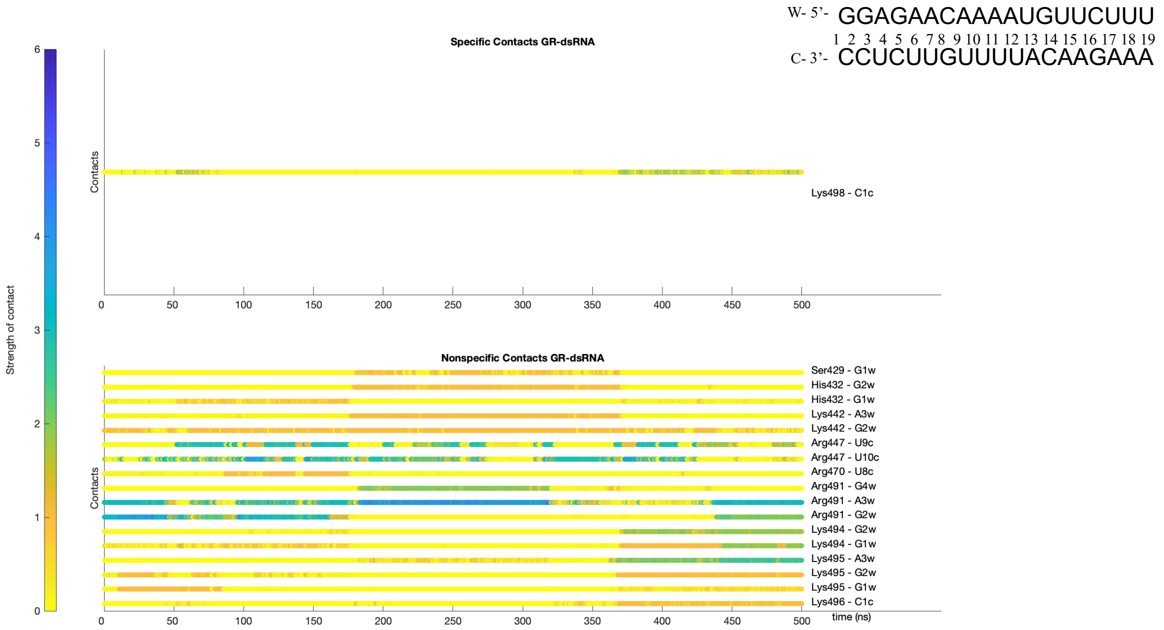

Figure S10: Dynamic contacts map for the specific and nonspecific GR-dsRNA interactions.

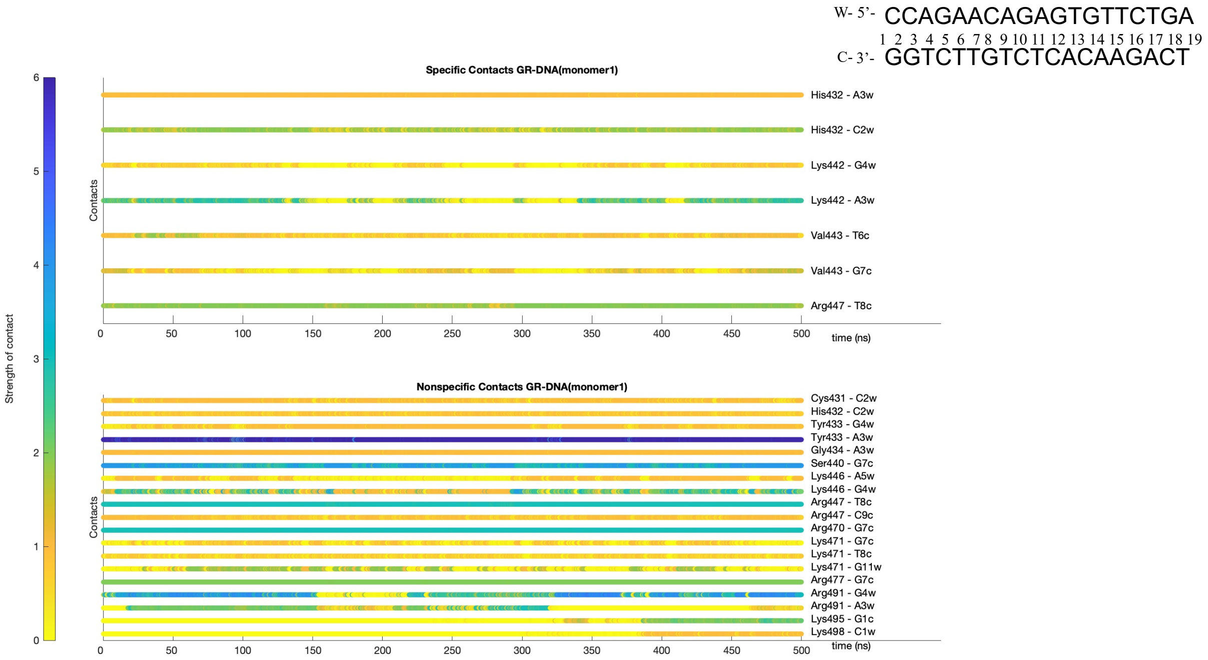

Figure S11: Dynamic contacts map for the specific and nonspecific GR(monomer1)-DNA interactions.

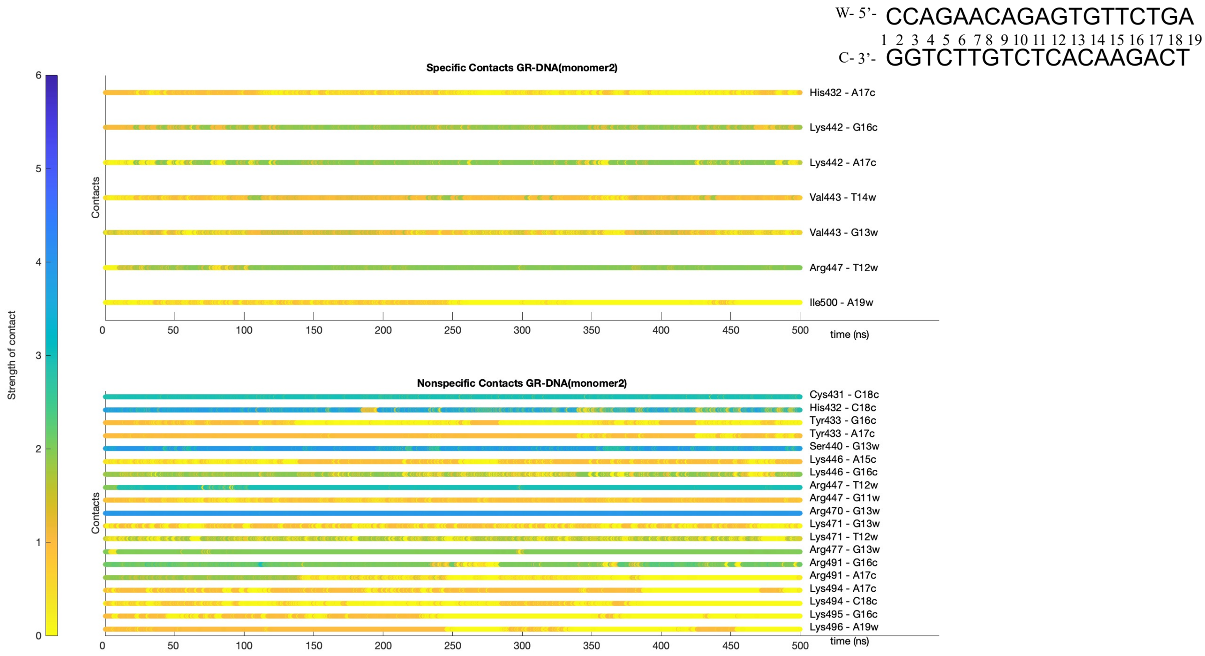

Figure S12: Dynamic contact map for the specific and nonspecific GR(monomer2)-DNA interactions.

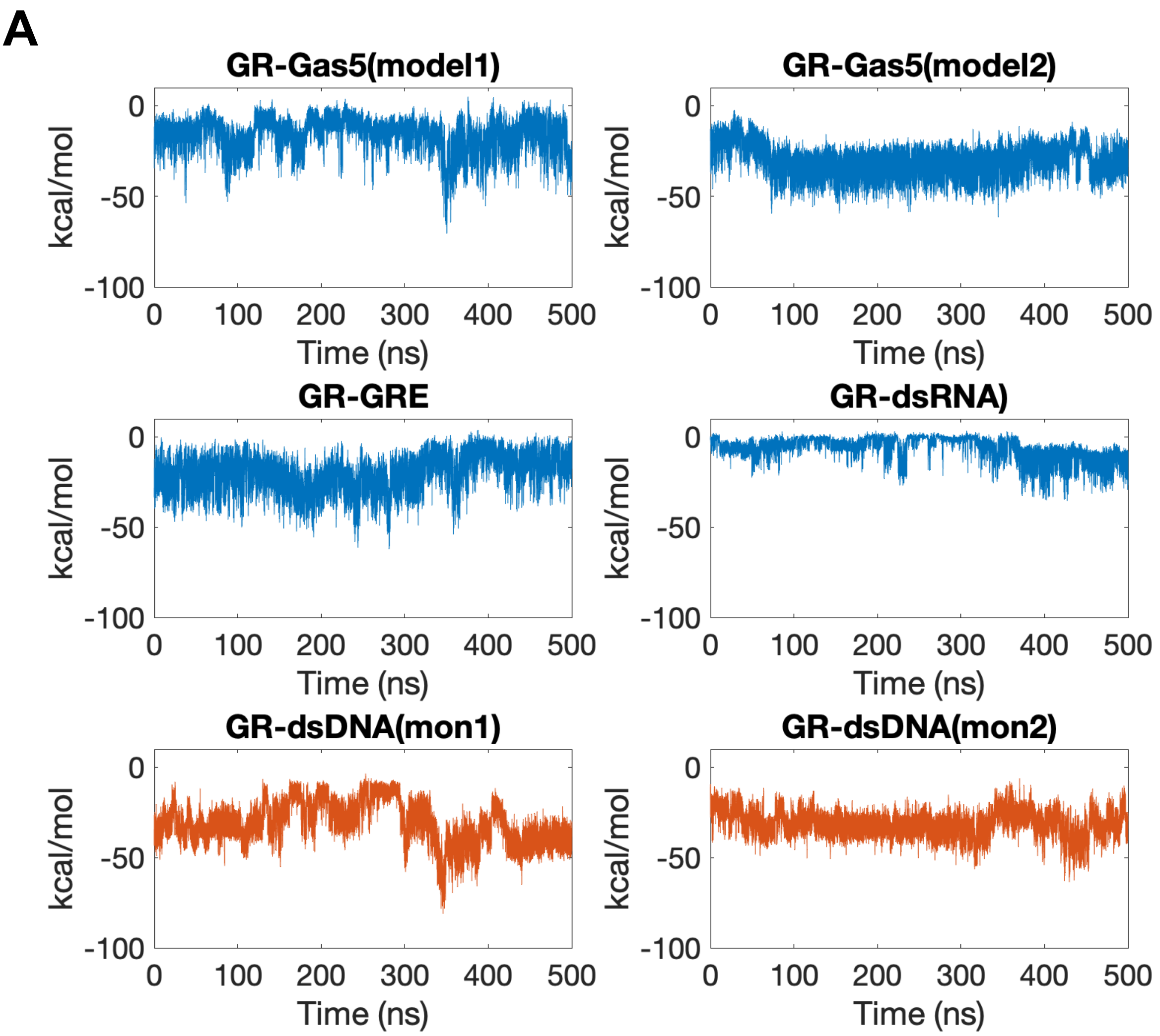
A
GR-Gas5(model1)
GR-Gas5(model2)
GR-GRE
GR-dsRNA)
GR-dsDNA(mon1)
GR-dsDNA(mon2)
kcal/mol
0
-50
-100
0
100
200
300
400
500
Time (ns)

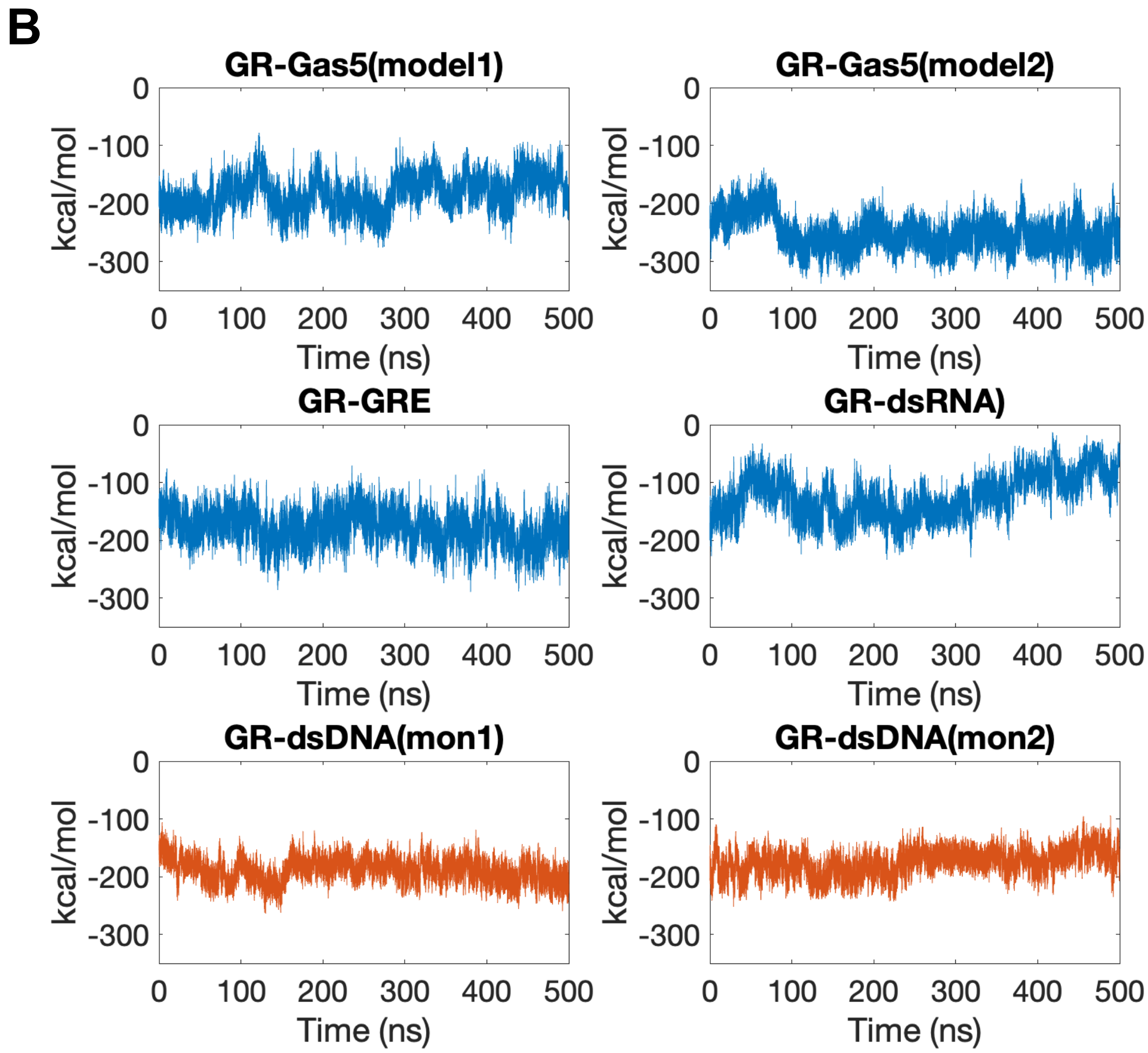

Figure S13: Specific (A) and nonspecific (B) GR-DNA/RNA interaction energies along the corresponding MD trajectories.

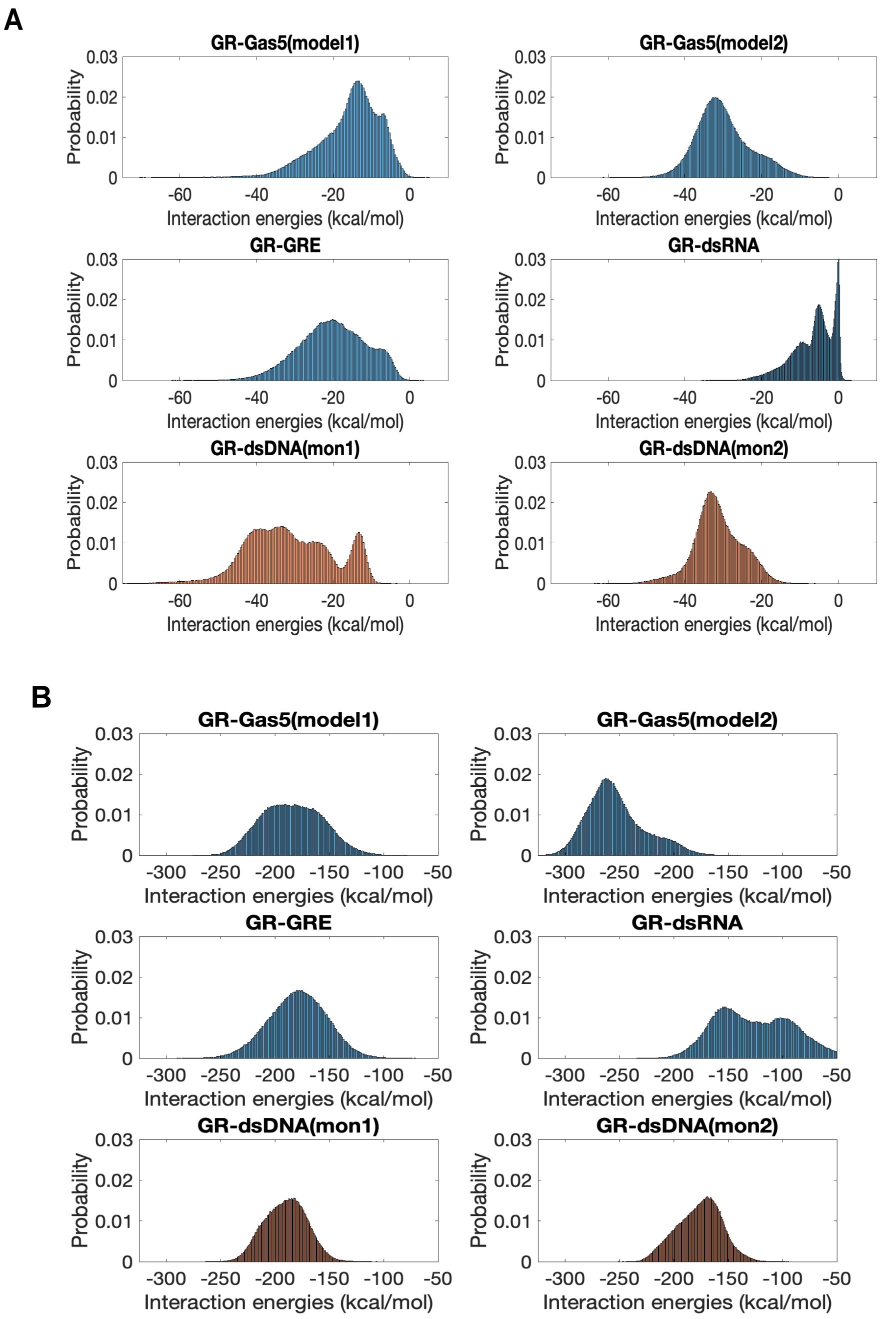

Figure S14: Specific (A) and nonspecific (B) GR-DNA/RNA interaction energies distributions

Table S1. Binding energies estimated by the MMPBSA and MMGBSA analyses for the GR-DNA/RNA complexes.

	MMPBSA (kcal/mol)	MMGBSA (kcal/mol)
GR-DNA	-197.2±17.7	-197.8±15.3
GR(mon1)-DNA	-99.8±14.0	-101.6±13.1
GR(mon2)-DNA	-97.1±13.1	-93.4±11.5
GR-dsRNA	-83.4±11.7	-72.9±9.7
GR-GRE_RNA	-70.3±14.1	-64.8±11.2
GR-Gas5_model1	-90.1±15.0	-79.7±13.1
GR-Gas5_model2	-130±16.6	-129.2±15.3

Table S2. Solvation energies estimated by the MMPBSA and MMGBSA analyses for the GR-DNA/RNA complexes.

	MMPBSA (kcal/mol)	MMGBSA (kcal/mol)
GR-DNA	8083.3±189.4	8082.7±188.3
GR(mon1)-DNA	4034.1±161.0	4032.2±159.7
GR(mon2)-DNA	4049.6±157.5	4053.1±157.0
GR-dsRNA	3877.3±193.7	3887.9±196.1
GR-GRE_RNA	3737.4±181.9	3743.0±181.6
GR-Gas5_model1	3896.3±225.9	3906.7±225.4
GR-Gas5_model2	4327.9±158.9	4328.5±159.5

Table S3. The polar and non-polar terms of the solvation energy calculated by the MMPBSA and MMGBSA analyses. The polar term represents the electrostatic interactions between the solute and the solvent continuum model. The non-polar term represents the hydrophobic effects and is estimated by a linear relation to the SASA.

	MMPBSA (kcal/mol)	MMGBSA (kcal/mol)
GR-DNA **polar**	8101.8±189.8	8101.2±188.7
GR-DNA **non-polar**	-18.5±1.2	
GR(mon1)-DNA **polar**	4043.2±161.9	4041.4±160.6
GR(mon1)-DNA **non-polar**	-9.1±1.4	
GR(mon2)-DNA **polar**	4059.0±157.9	4062.5±157.5
GR(mon2)-DNA **non-polar**	-9.4±0.8	
GR-dsRNA **polar**	3882.9±193.9	3893.5±196.2
GR-dsRNA **non-polar**	-5.6±0.7	
GR-GRE_RNA **polar**	3744.4±182.2	3750.0±181.9
GR-GRE_RNA **non-polar**	-7.0±0.8	
GR-Gas5_model1 **polar**	3904.7±226.2	3915.1±225.6
GR-Gas5_model1 **non-polar**	-8.38±0.9	
GR-Gas5_model2 **polar**	4339.1±159.4	4339.76±160.0
GR-Gas5_model2 **non-polar**	-11.3±0.8	

A

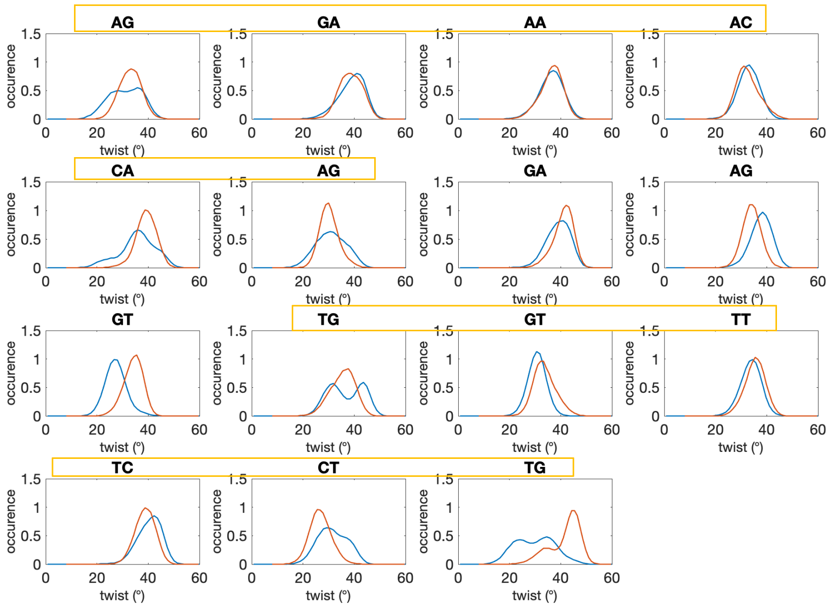
AG
GA
AA
AC
CA
AG
GA
AG
GT
TG
GT
TT
TC
CT
TG
occurence
twist (°)
0
20
40
60
0
0.5
1
1.5

B

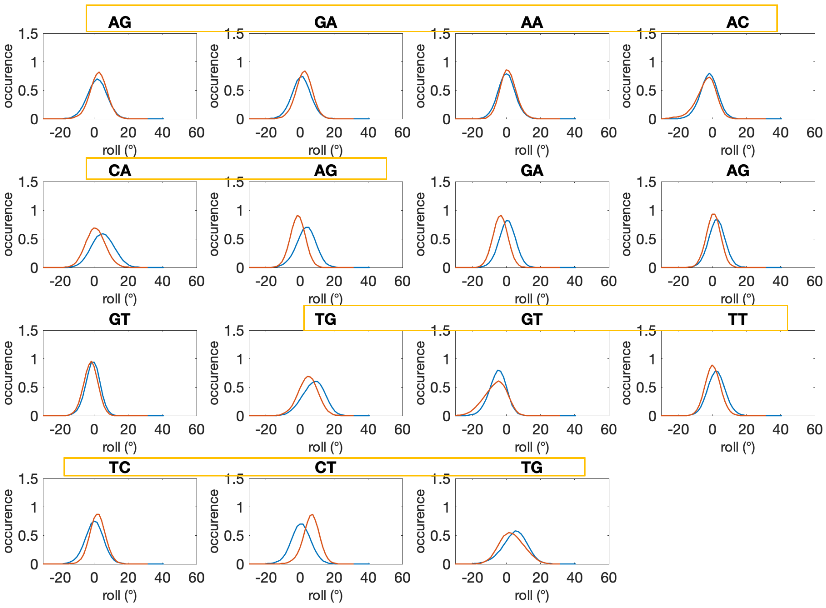
AG
GA
AA
AC
CA
AG
GA
AG
GT
TG
GT
TT
TC
CT
TG
occurence
roll (°)
-20
0
20
40
60
0
0.5
1
1.5

C

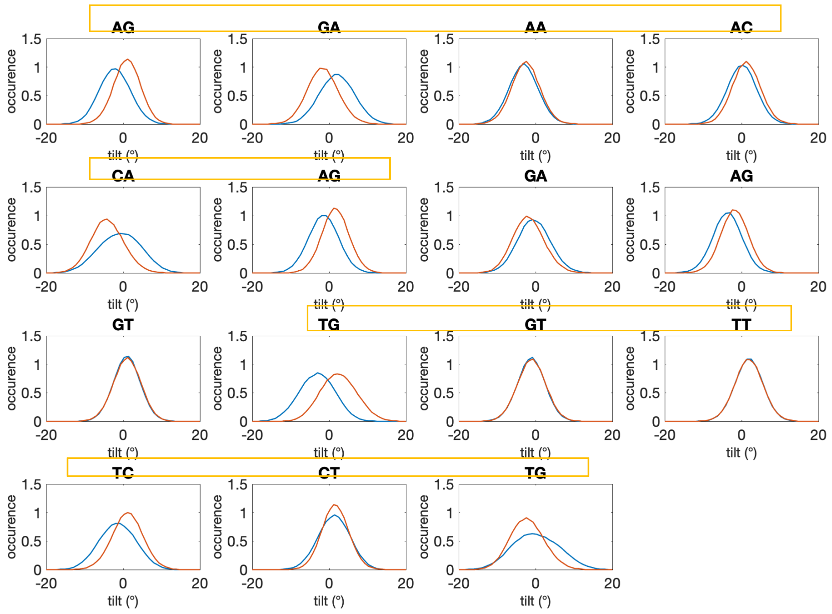
AG
GA
AA
AC
CA
AG
GA
AG
GT
TG
GT
TT
TC
CT
TG
occurence
tilt (°)
1.5
1
0.5
0
-20
0
20

D

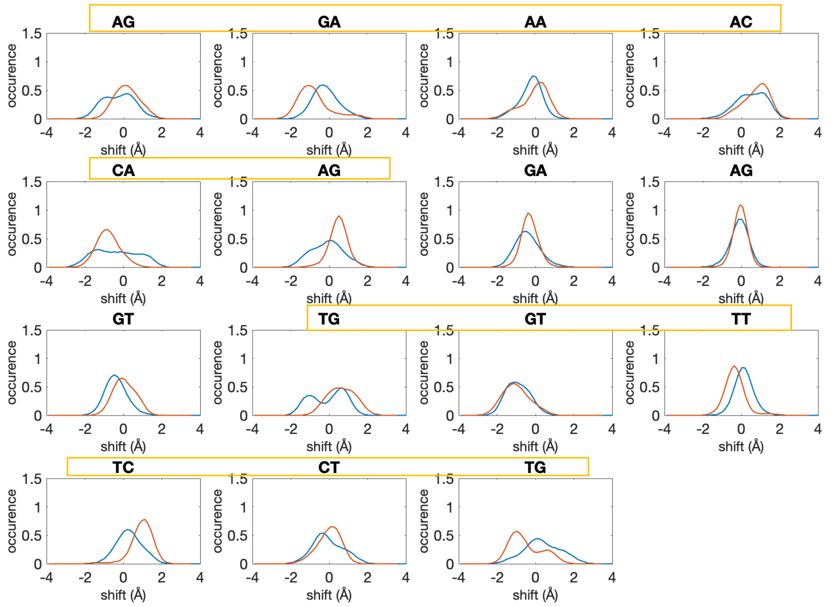
AG
GA
AA
AC
CA
AG
GA
AG
GT
TG
GT
TT
TC
CT
TG
occurence
shift (Å)
1.5
1
0.5
0
-4
-2
0
2
4

E

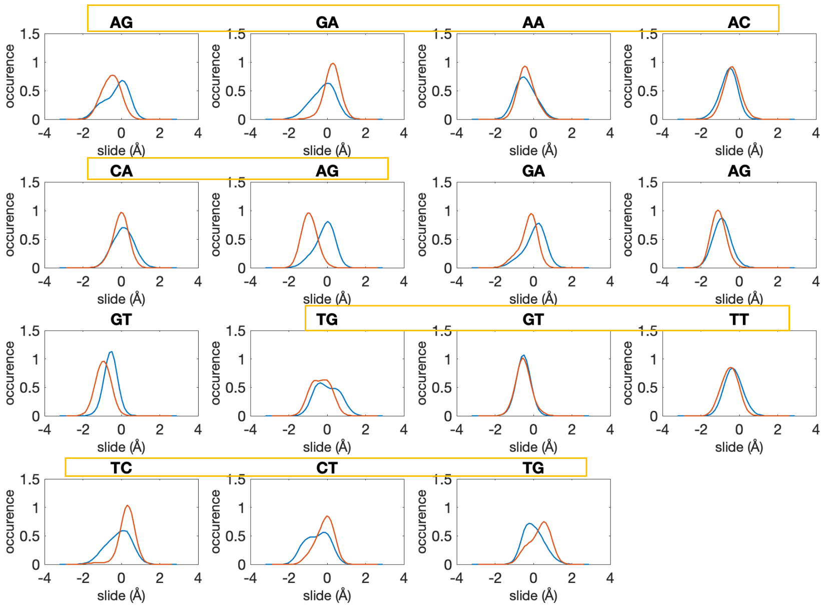

F

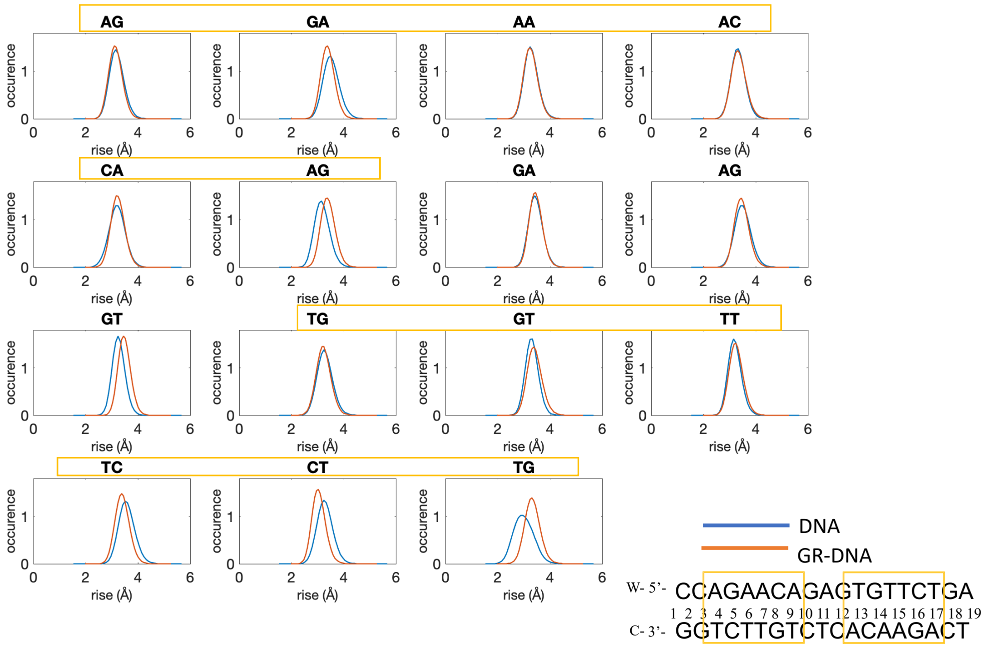

Figure S15: Helical parameters distributions for unbound and GR-bound DNA. **A.** Twist **B.** Roll **C.** Tilt **D.** Shift E. Slide **F.** Rise. The glucocorticoid receptor response elements 1 and 2 are highlighted with yellow.

A

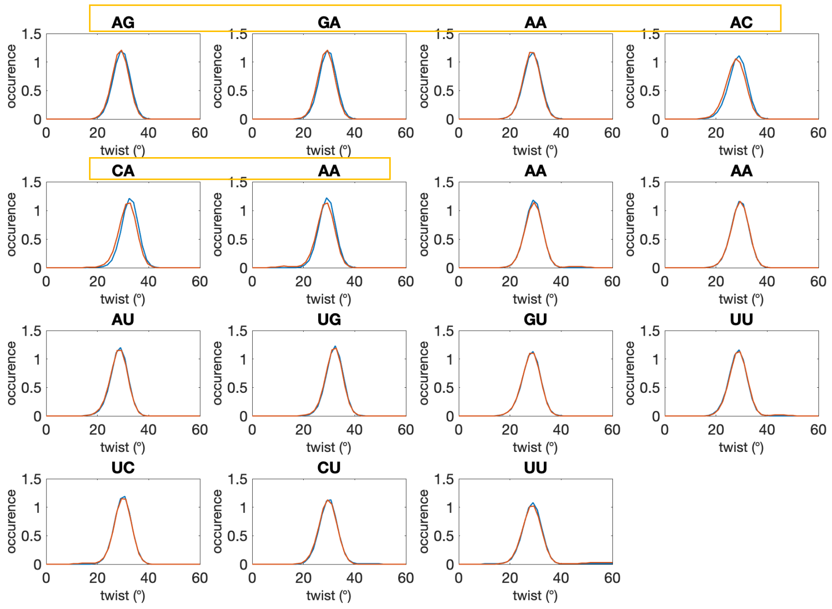
AG
GA
AA
AC
CA
AA
AA
AA
AU
UG
GU
UU
UC
CU
UU
occurence
twist (°)

B

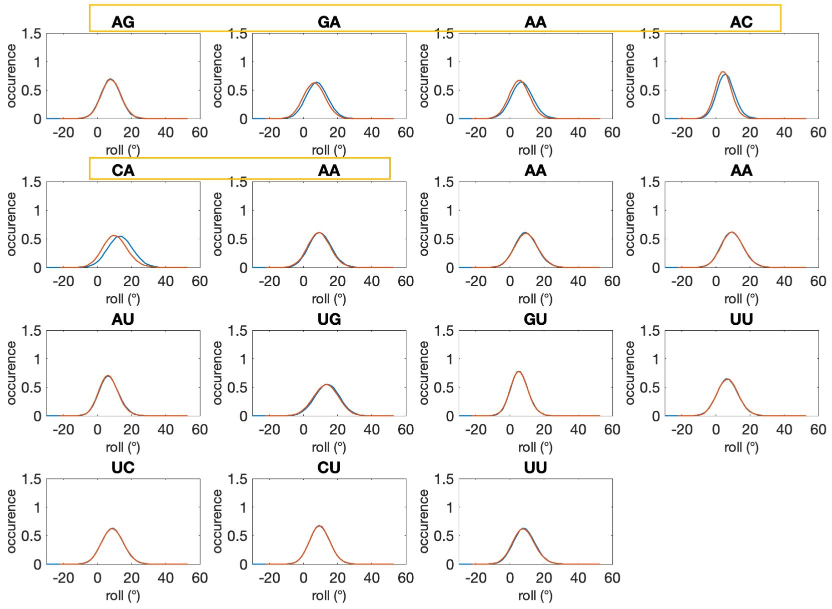
AG
GA
AA
AC
CA
AA
AA
AA
AU
UG
GU
UU
UC
CU
UU
occurence
roll (°)

C

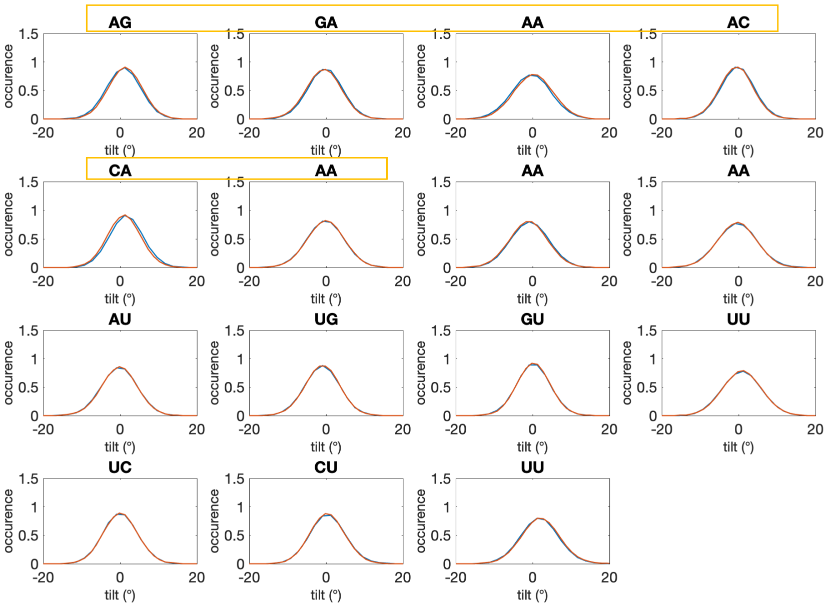
AG
GA
AA
AC
CA
AA
AA
AA
AU
UG
GU
UU
UC
CU
UU
occurence
tilt (°)

D

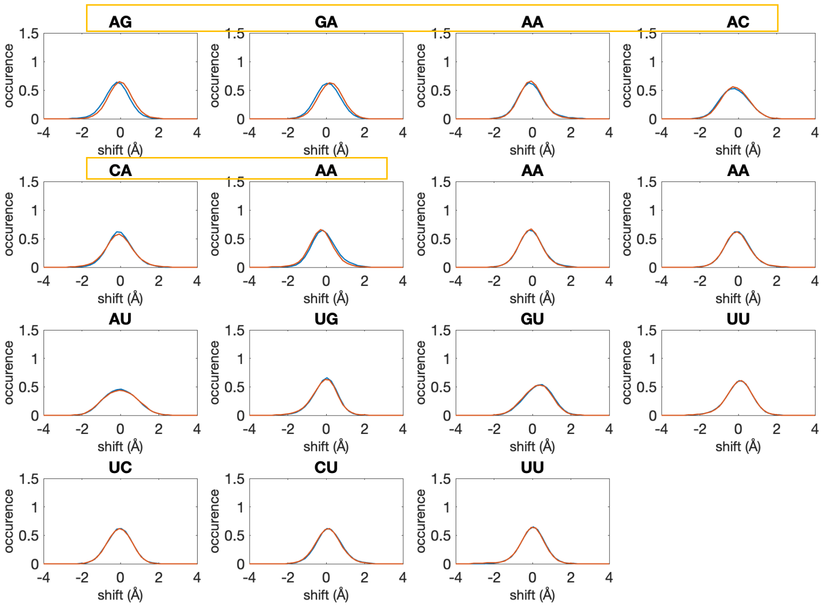
AG
GA
AA
AC
CA
AA
AA
AA
AU
UG
GU
UU
UC
CU
UU
occurence
shift (Å)

E

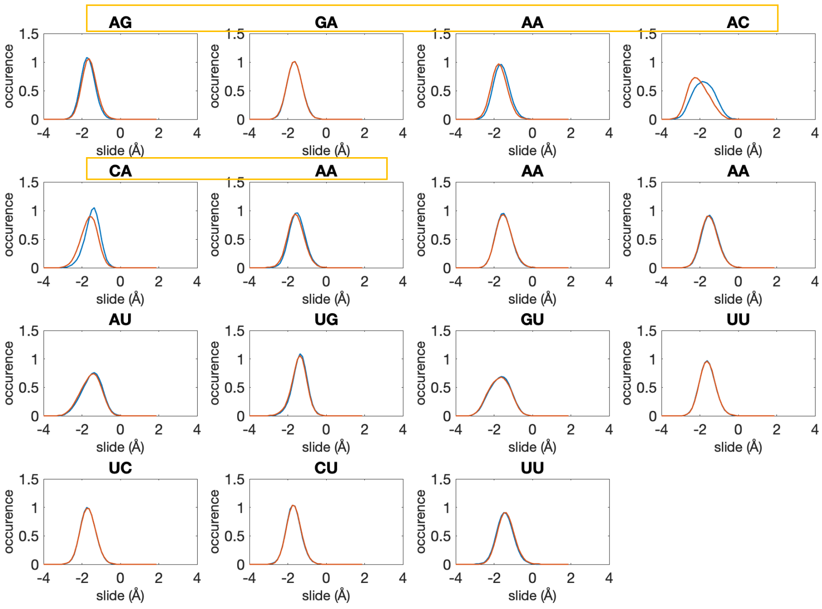

F

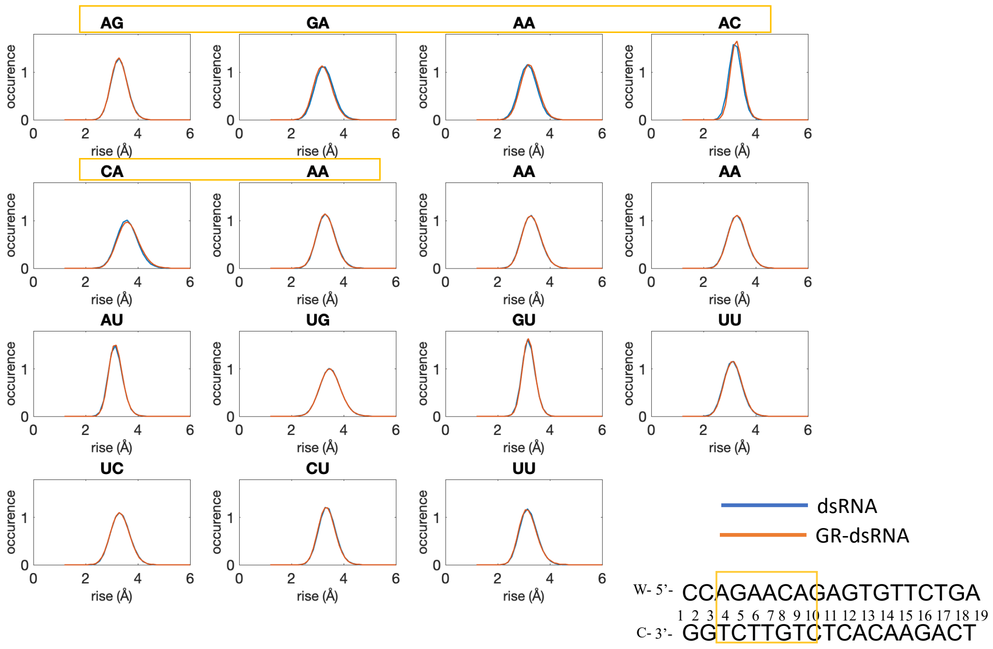

Figure S16: Helical parameters distributions for unbound and GR-bound dsRNA. **A.** Twist **B.** Roll **C.** Tilt **D.** Shift E. Slide **F.** Rise. The glucocorticoid receptor response element is highlighted with yellow.

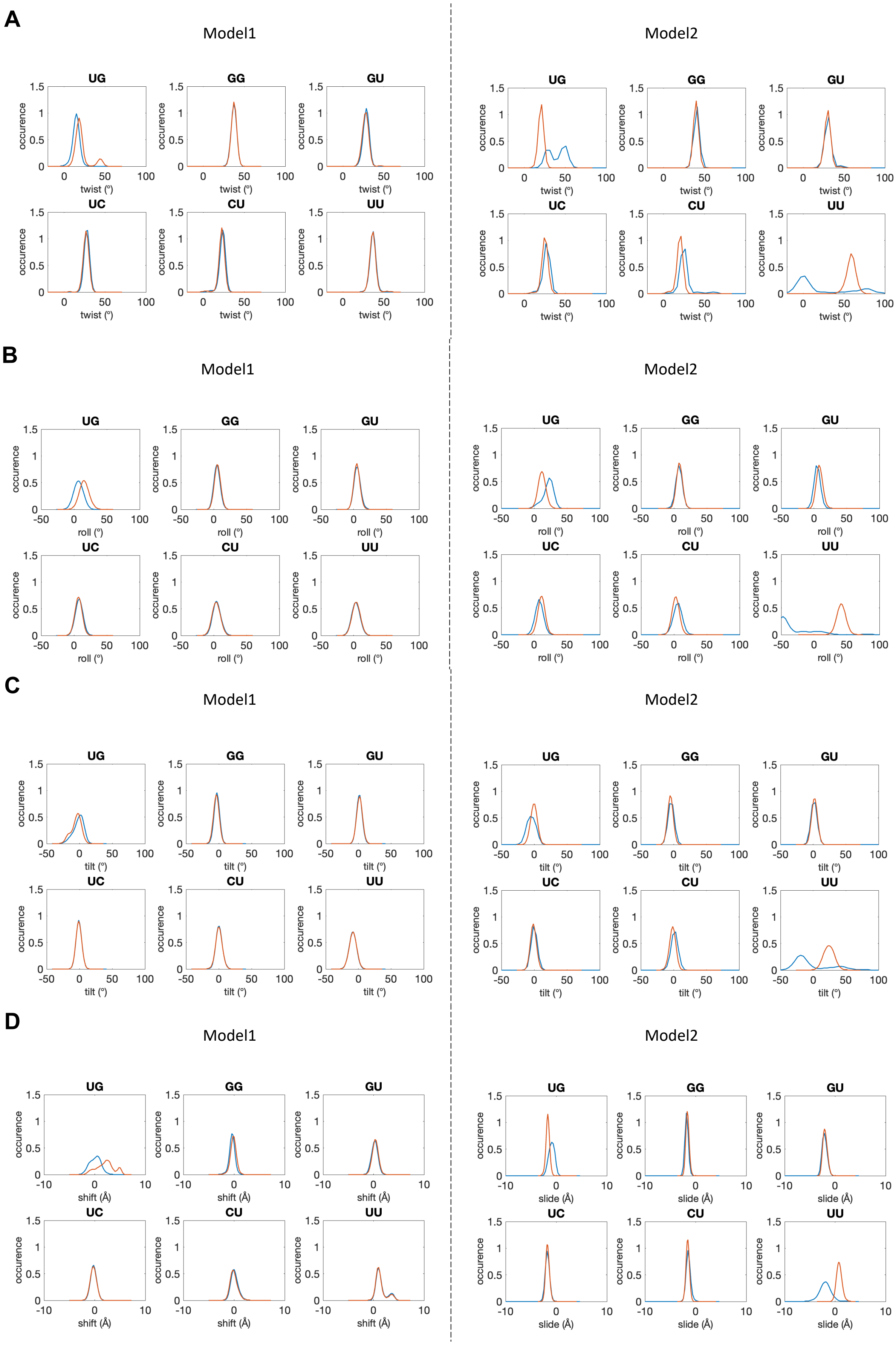
A
Model1
UG
GG
GU
UC
CU
UU
occurence
twist (°)
Model2
UG
GG
GU
UC
CU
UU
occurence
twist (°)
B
Model1
UG
GG
GU
UC
CU
UU
occurence
roll (°)
Model2
UG
GG
GU
UC
CU
UU
occurence
roll (°)
C
Model1
UG
GG
GU
UC
CU
UU
occurence
tilt (°)
Model2
UG
GG
GU
UC
CU
UU
occurence
tilt (°)
D
Model1
UG
GG
GU
UC
CU
UU
occurence
shift (Å)
Model2
UG
GG
GU
UC
CU
UU
occurence
slide (Å)

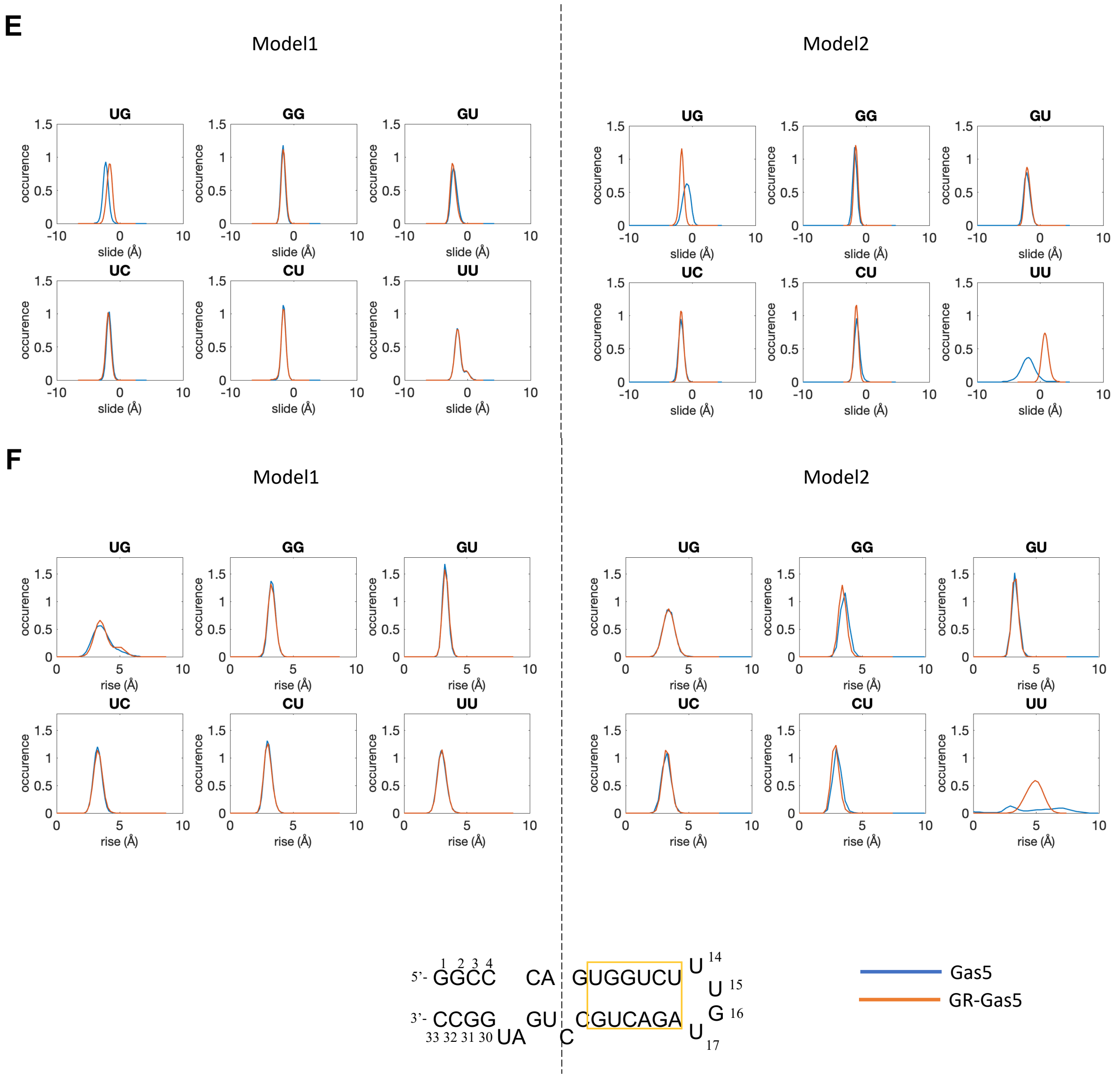

Figure S17: Helical parameters distributions of the glucocorticoid receptor response element for unbound and GR-bound Gas5_RNA for model1 (left panels) and model2 (right panels) . **A.** Twist **B.** Roll **C.** Tilt **D.** Shift E. Slide **F.** Rise.

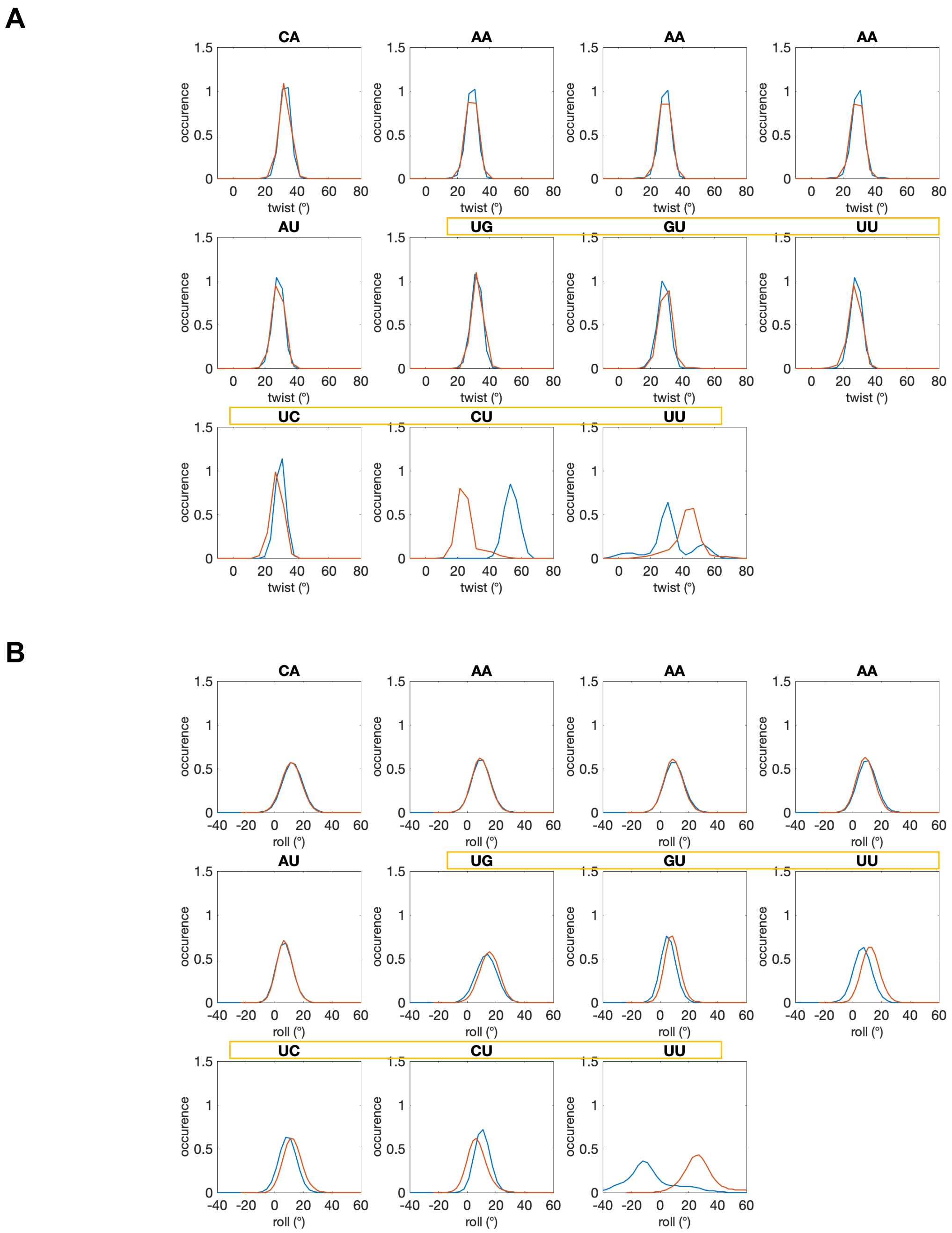
A
CA
AA
AA
AA
AU
UG
GU
UU
UC
CU
UU
occurence
twist (°)
B
CA
AA
AA
AA
AU
UG
GU
UU
UC
CU
UU
occurence
roll (°)

C

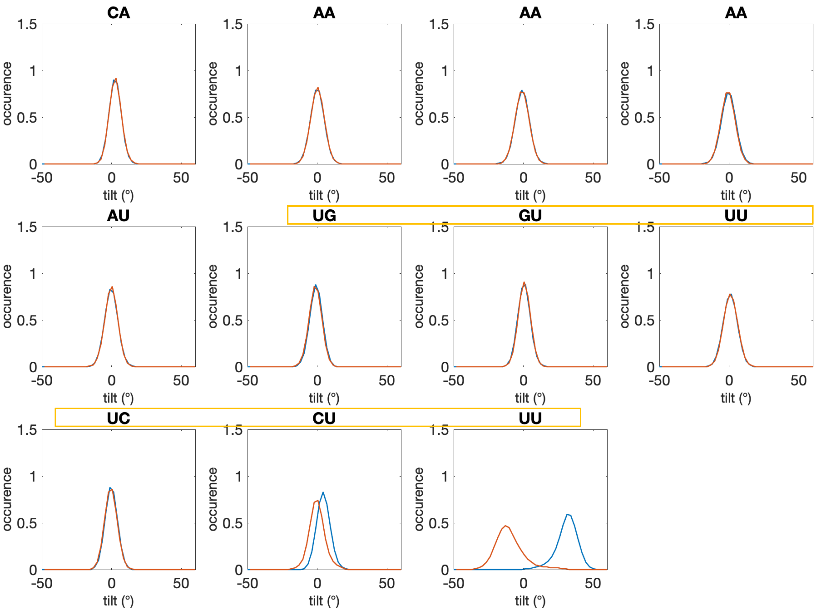
CA
AA
AA
AA
AU
UG
GU
UU
UC
CU
UU
occurence
tilt (°)
1.5
1
0.5
0
-50
0
50

D

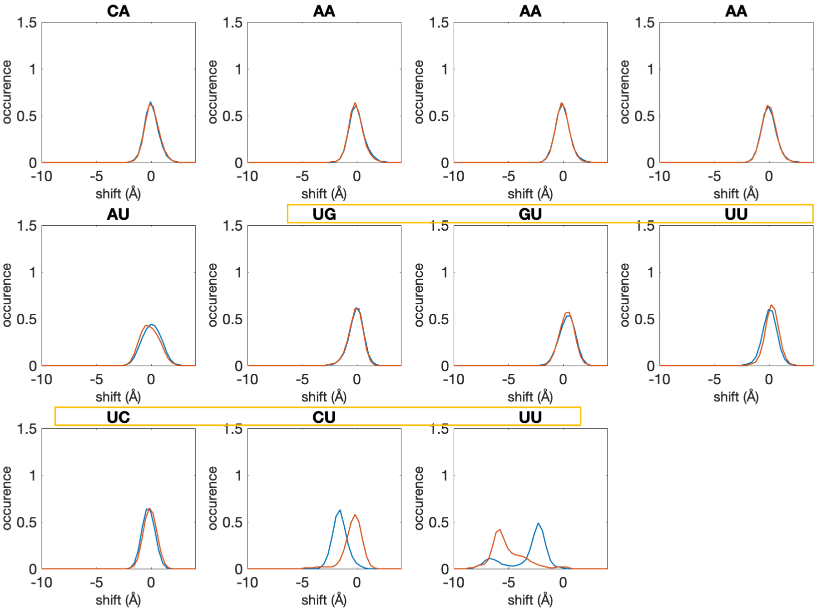
CA
AA
AA
AA
AU
UG
GU
UU
UC
CU
UU
occurence
shift (Å)
1.5
1
0.5
0
-10
-5
0

E

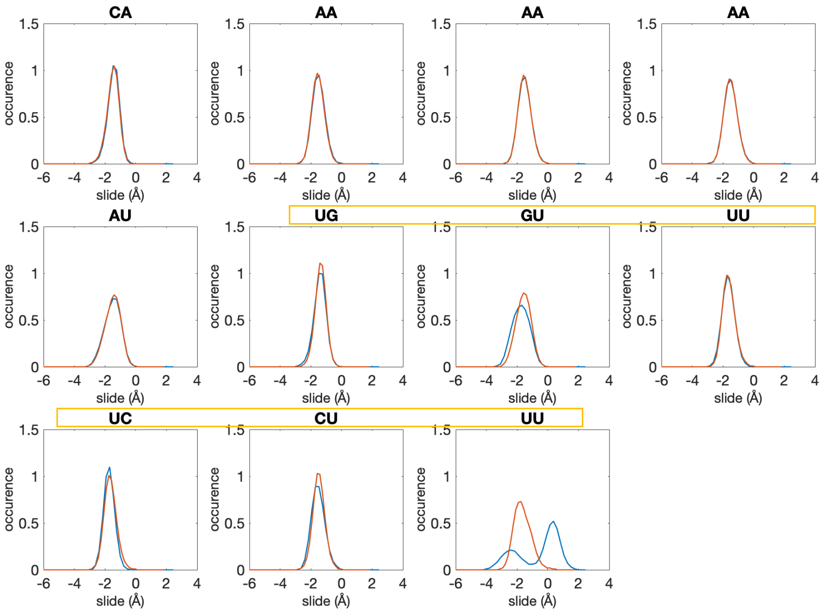

F

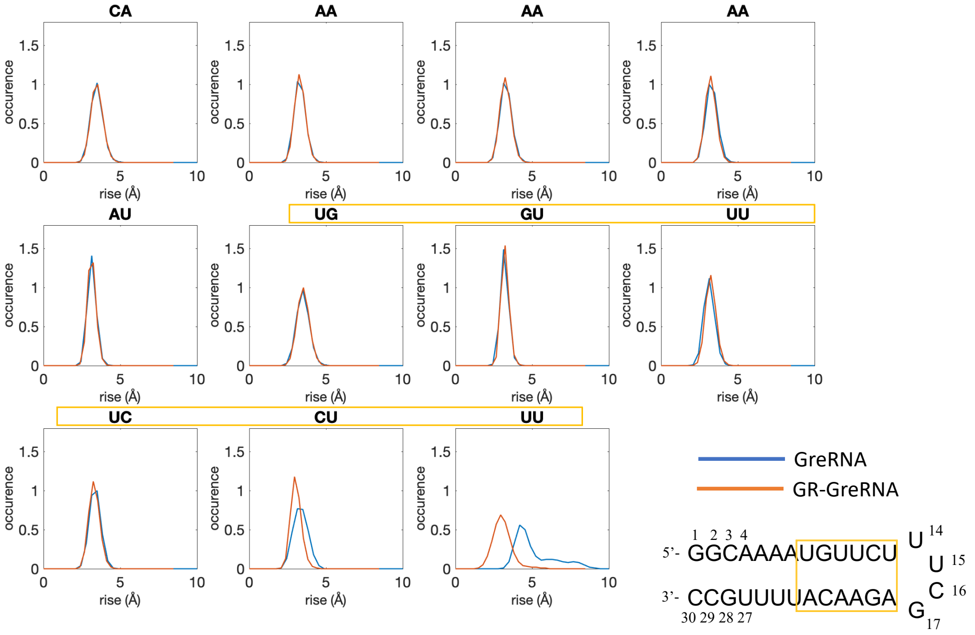

Figure S18: Helical parameters distributions for unbound and GR-bound GRE_RNA. **A.** Twist **B.** Roll **C.** Tilt **D.** Shift E. Slide **F.** Rise. The glucocorticoid receptor response element is highlighted with yellow.

A

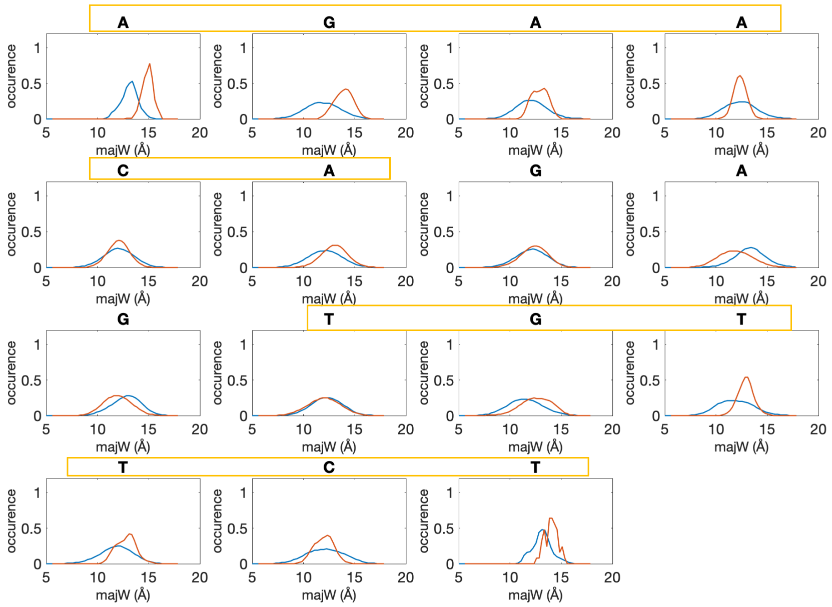
A
G
A
A
C
A
G
A
G
T
G
T
T
C
T
occurence
majW (Å)

B

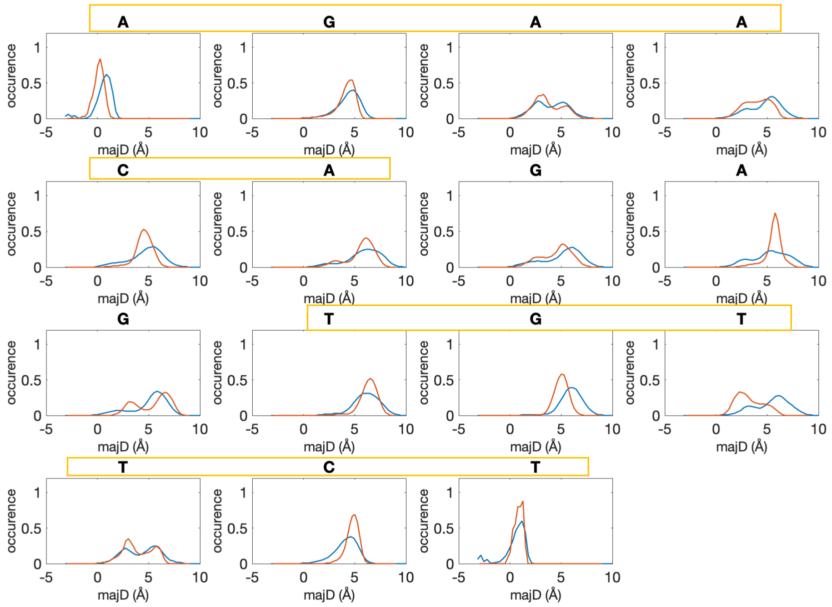
A
G
A
A
C
A
G
A
G
T
G
T
T
C
T
occurence
majD (Å)

C

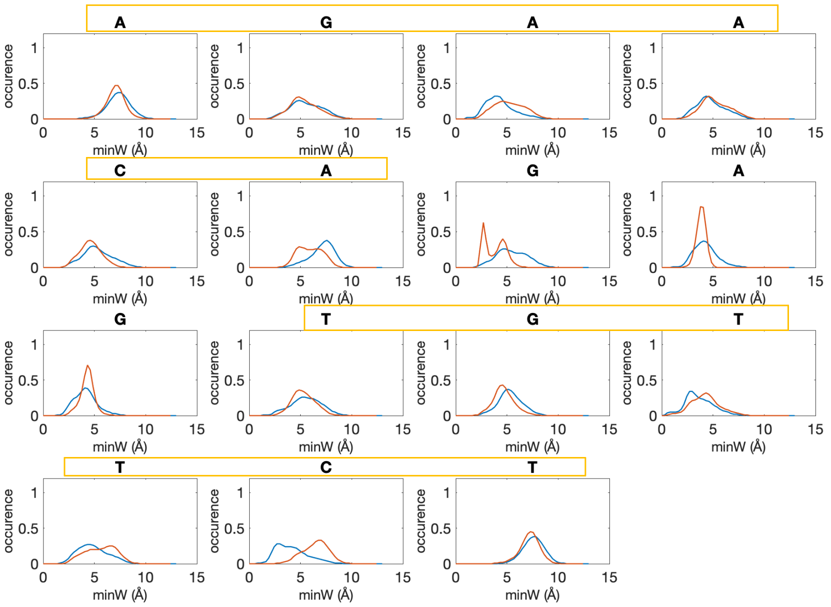

D

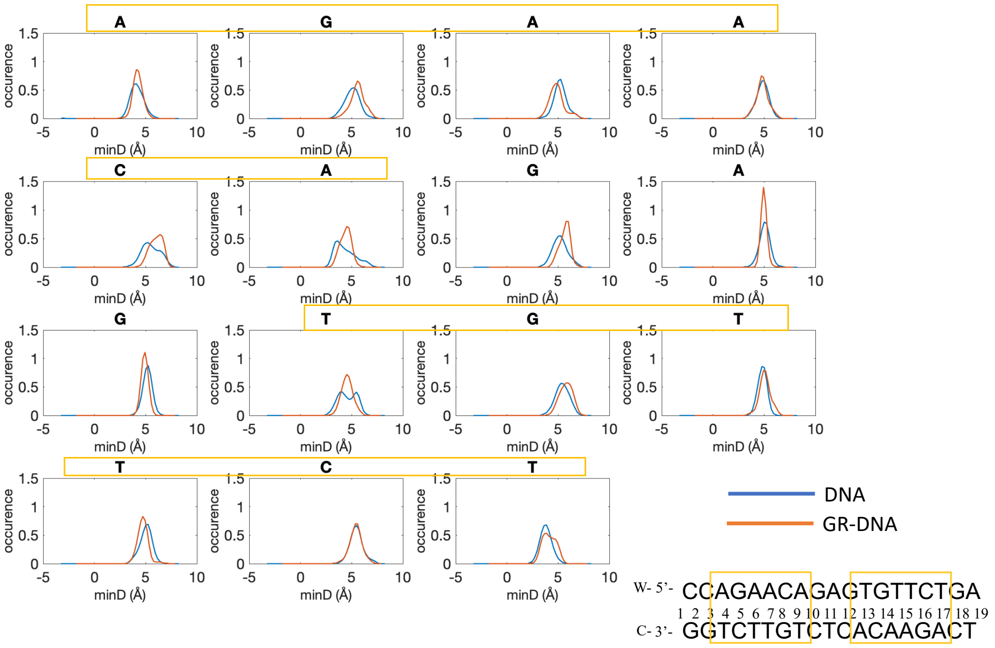

Figure S19: Groove parameters distributions for unbound and GR-bound DNA. **A.** Major groove width **B.** Major groove depth **C.** Minor groove width **D.** Minor groove depth. The glucocorticoid receptor response elements 1 and 2 are highlighted with yellow.

A

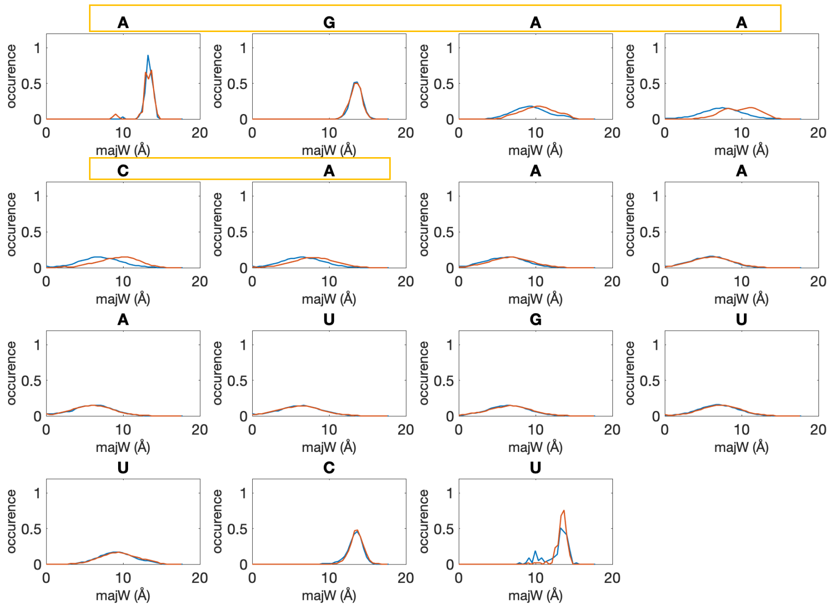
A
G
A
A
C
A
A
A
A
U
G
U
U
C
U
occurence
majW (Å)

B

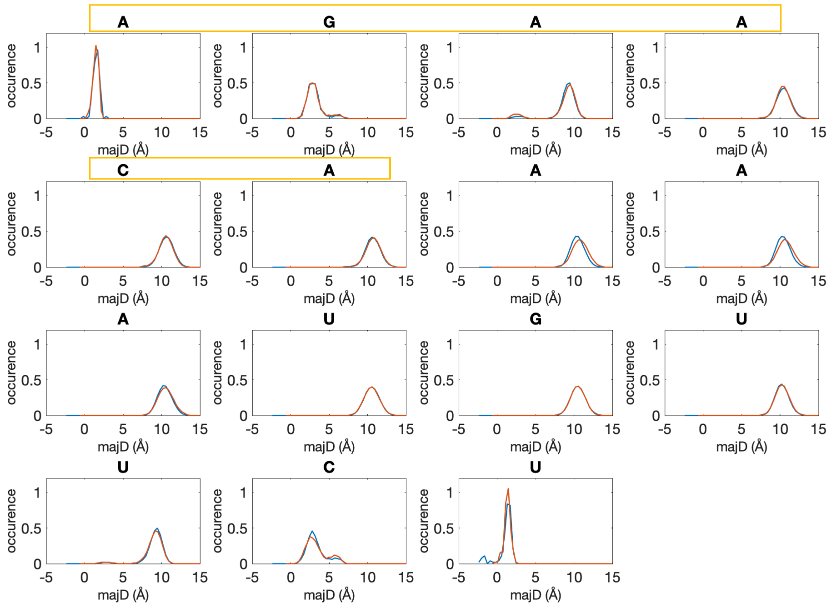
A
G
A
A
C
A
A
A
A
U
G
U
U
C
U
occurence
majD (Å)

C

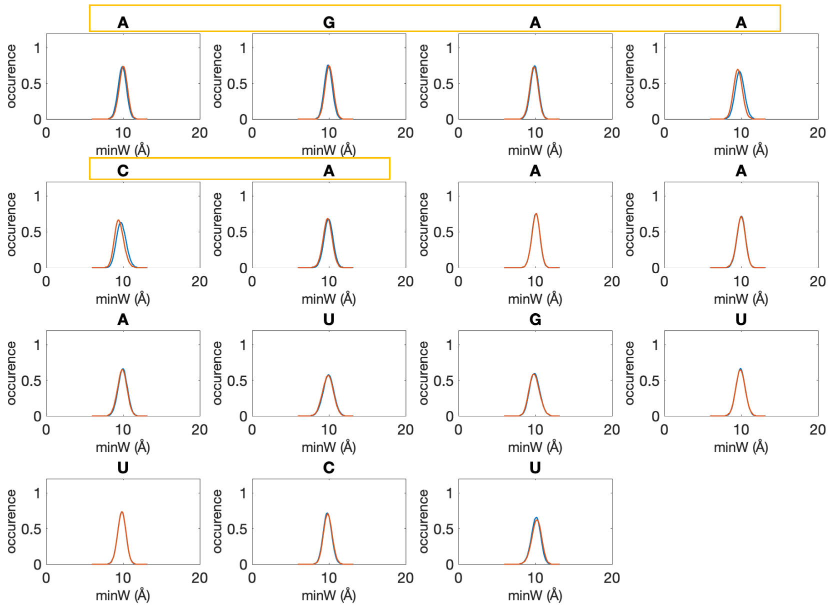

D

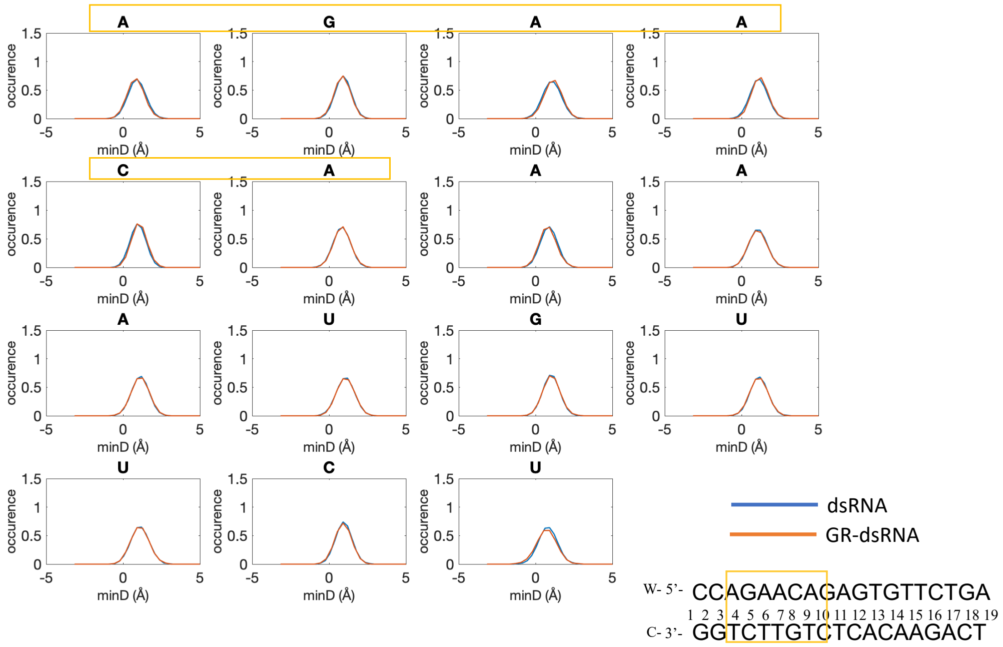

Figure S20: Groove parameters distributions for unbound and GR-bound dsRNA. **A.** Major groove width **B.** Major groove depth **C.** Minor groove width **D.** Minor groove depth. The glucocorticoid receptor response element is highlighted with yellow.

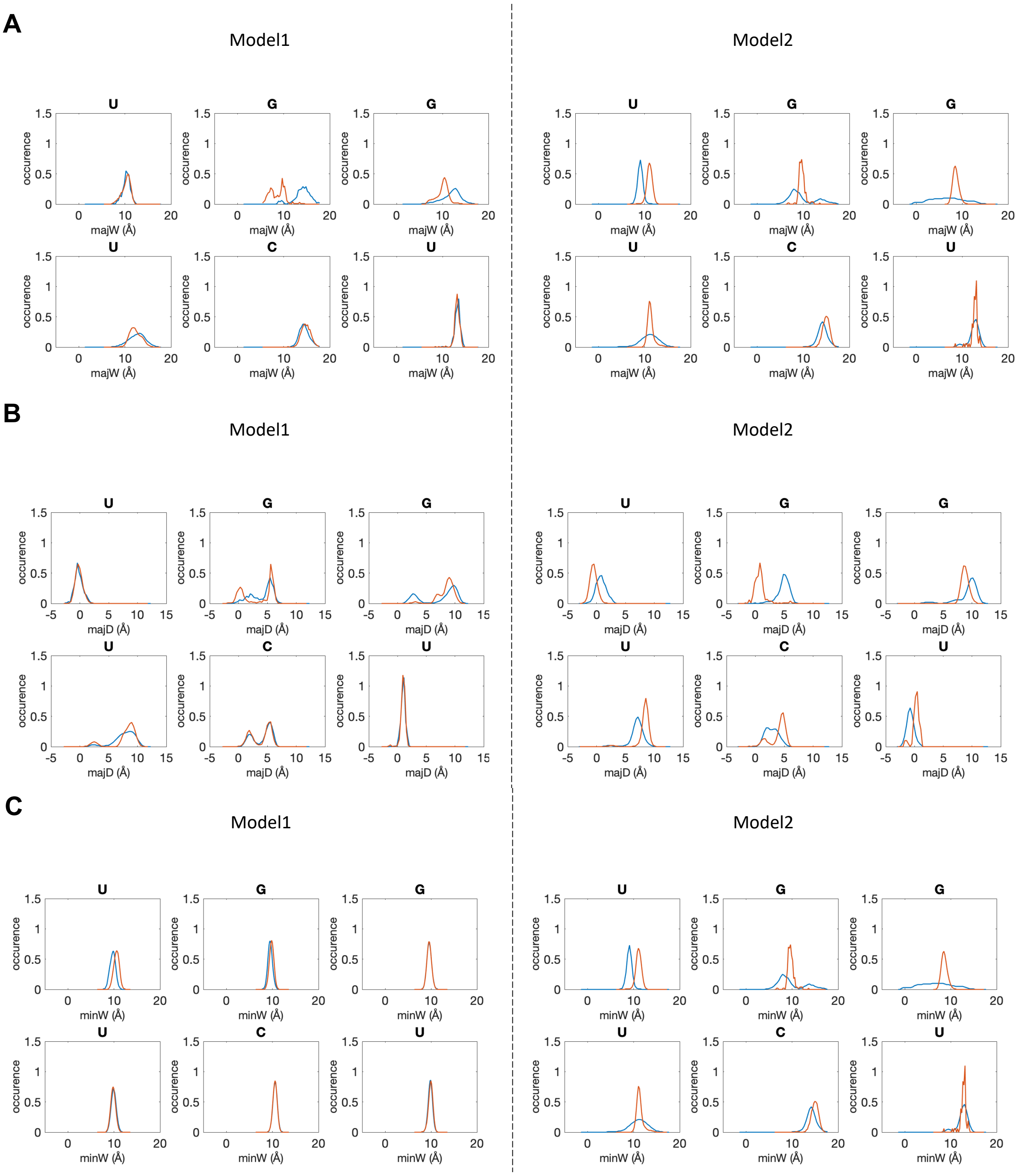

A
Model1
Model2
U
G
G
U
C
U
occurence
majW (Å)
B
Model1
Model2
majD (Å)
C
Model1
Model2
minW (Å)

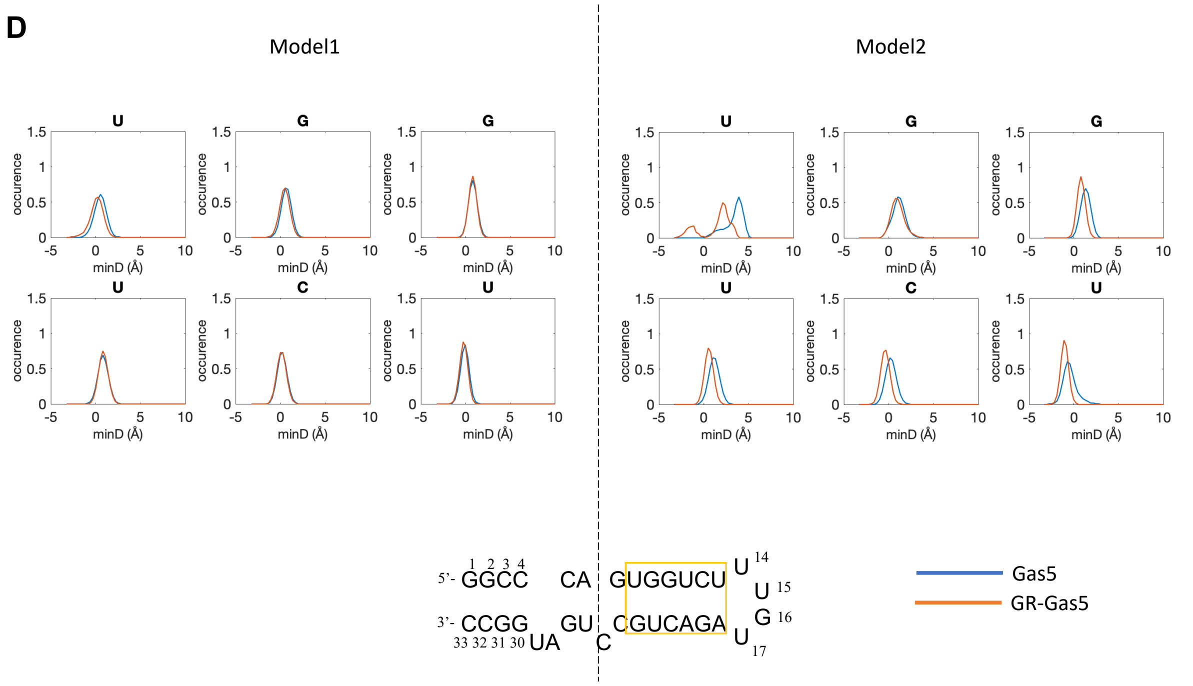

Figure S21: Groove parameters distributions of the glucocorticoid receptor response element for unbound and GR-bound Gas5_RNA. **A.** Major groove width **B.** Major groove depth **C.** Minor groove width **D.**

A

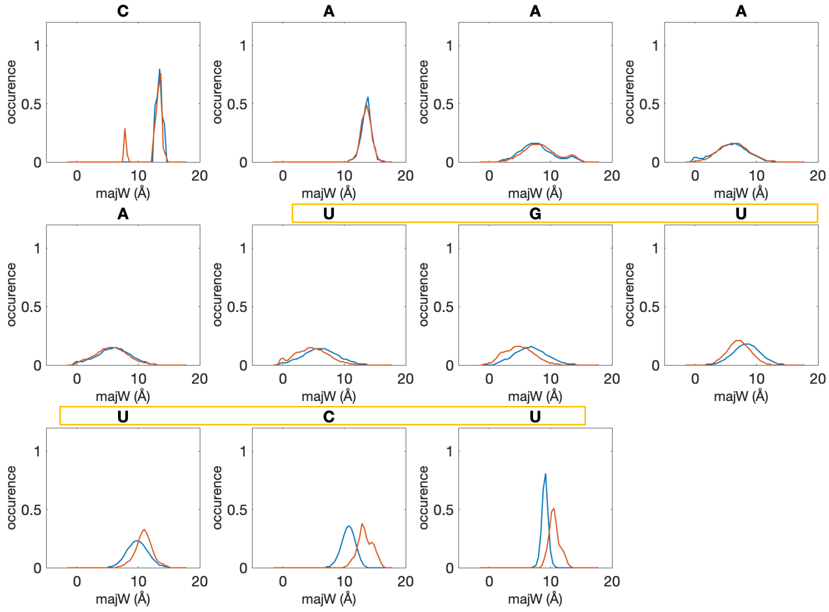
C
A
A
A
A
U
G
U
U
C
U
occurence
majW (Å)

B

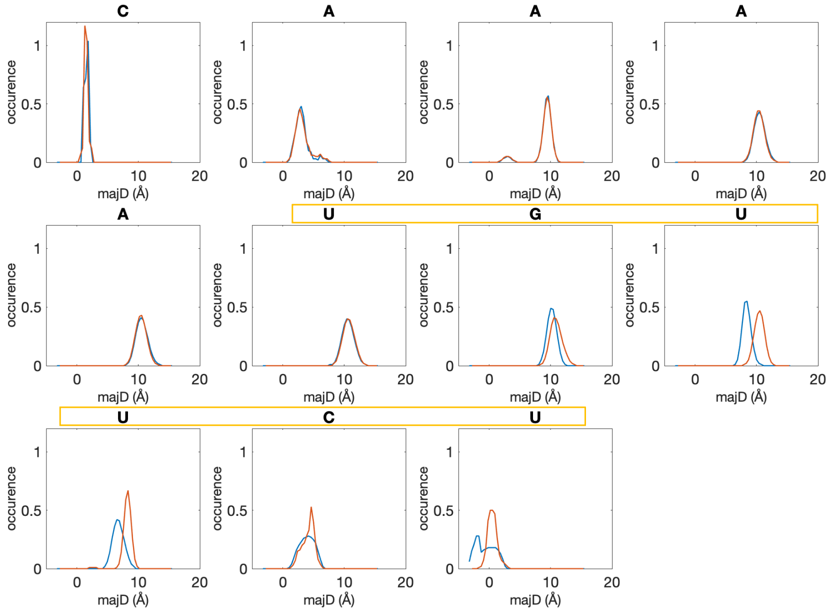
C
A
A
A
A
U
G
U
U
C
U
occurence
majD (Å)

C

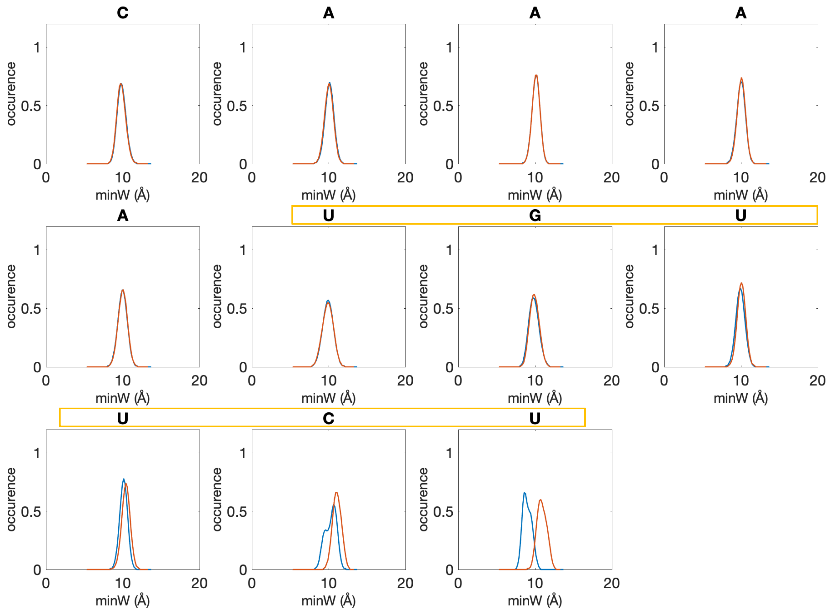

D

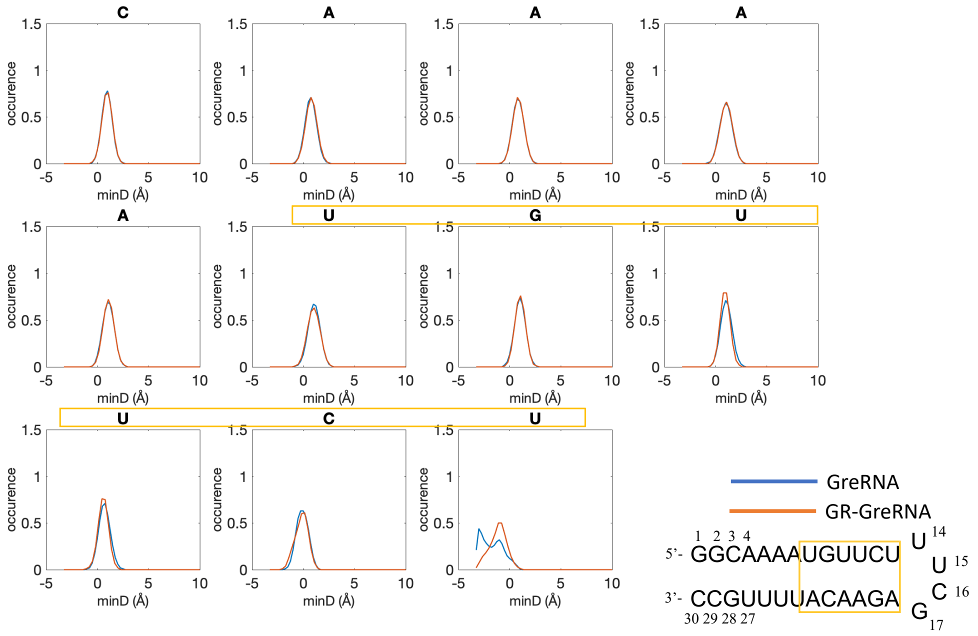

Figure S22: Groove parameters distributions for unbound and GR-bound GRE_RNA. **A.** Major groove width **B.** Major groove depth **C.** Minor groove width **D.** Minor groove depth. The glucocorticoid receptor response element is highlighted with yellow.

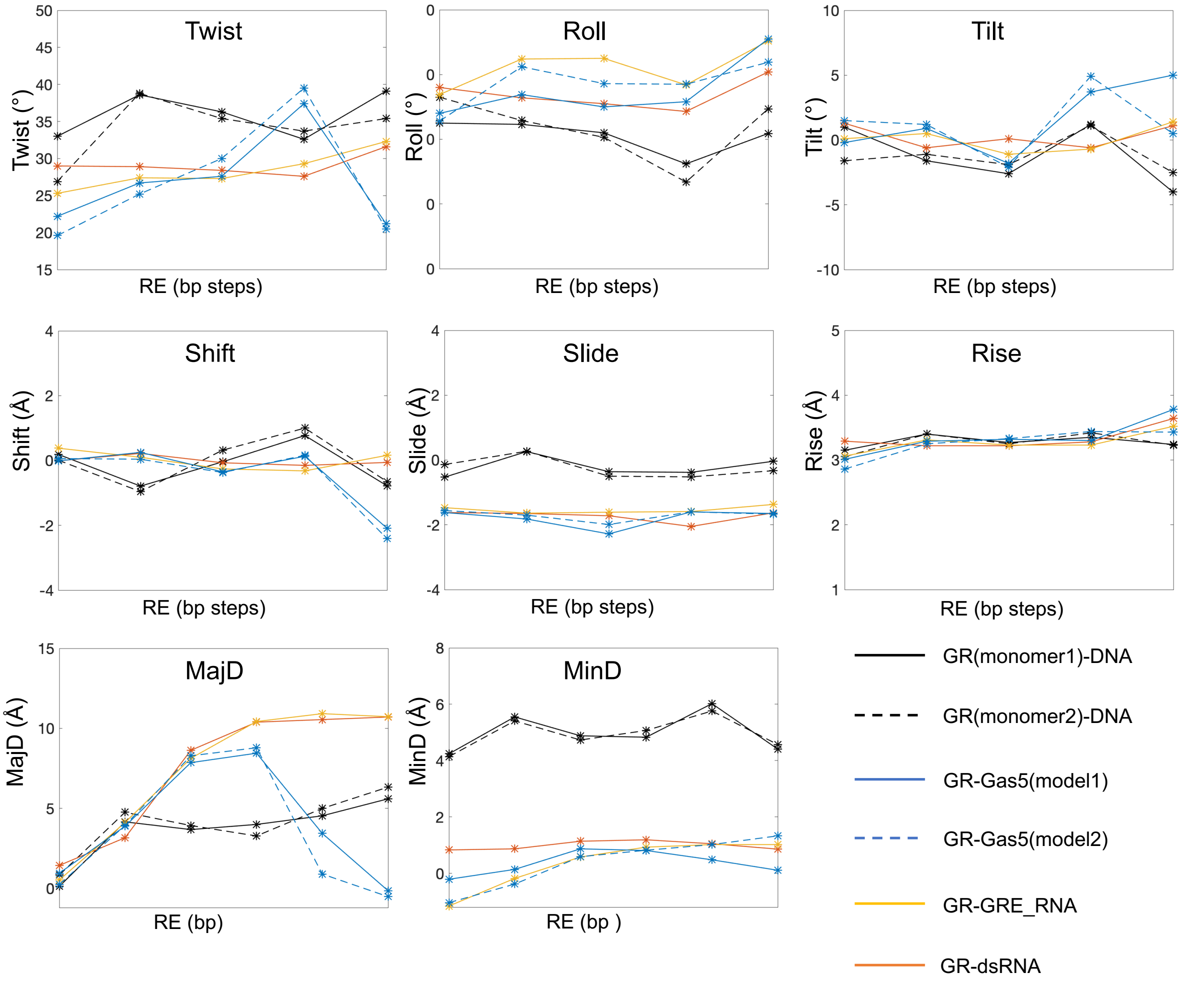

Figure S23: Comparison of average values for helical parameters and groove depths in different nucleic acids model systems for the b.p. within the GRE-site for the GR-bound systems.

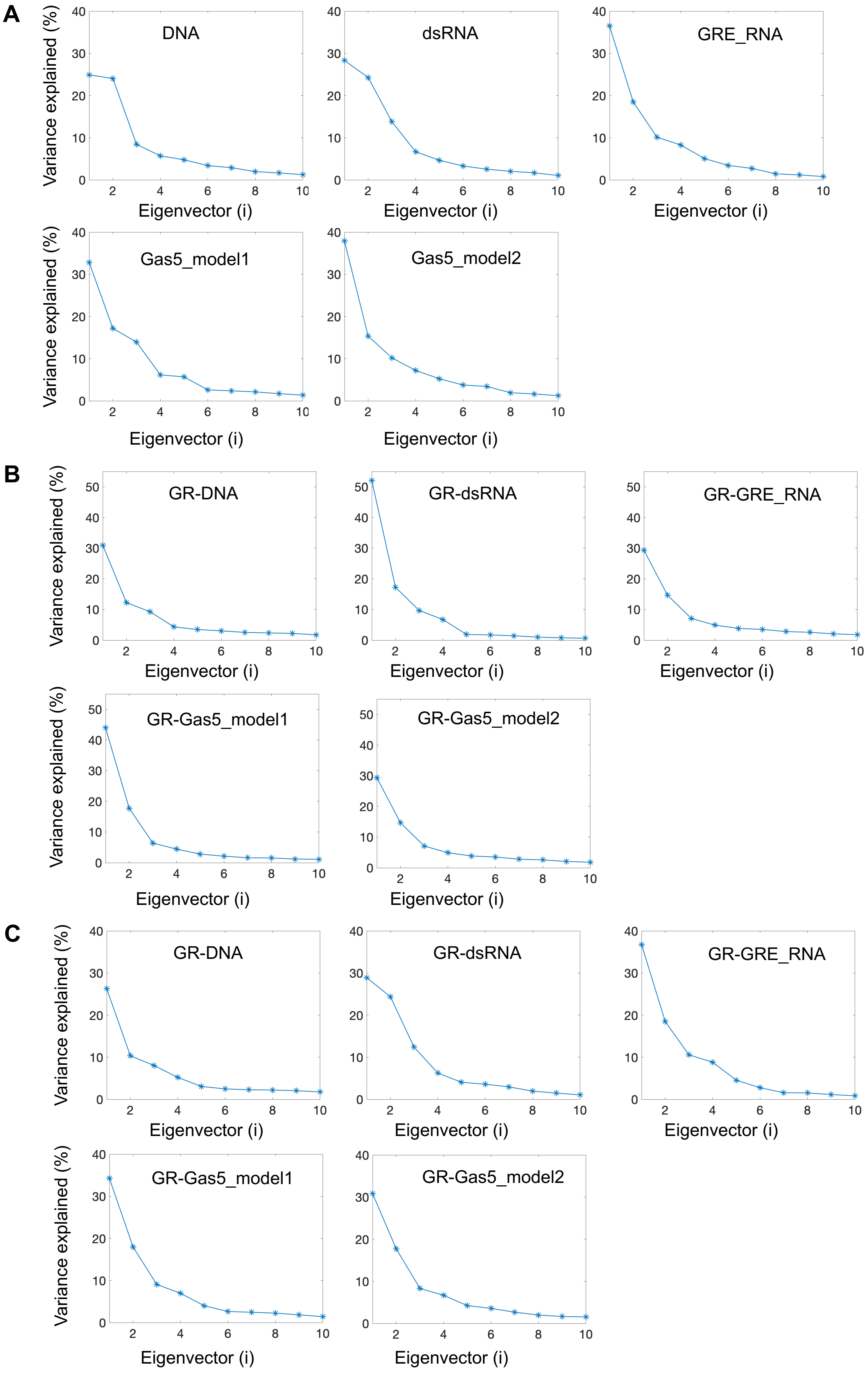
A
DNA
dsRNA
GRE_RNA
Gas5_model1
Gas5_model2
B
GR-DNA
GR-dsRNA
GR-GRE_RNA
GR-Gas5_model1
GR-Gas5_model2
C
GR-DNA
GR-dsRNA
GR-GRE_RNA
GR-Gas5_model1
GR-Gas5_model2
Variance explained (%)
Eigenvector (i)

Figure S24: PCA analysis showing the variance explained by the first 10 eigenvectors. **A.** For unbound nucleic acids systems. For the GR-bound nucleic acids systems for the two PCA analyses performed: **B.** for the heavy atoms of the protein-DNA/RNA systems with the superposition on the nucleic acids and **C.** for heavy atoms of DNA/RNA with the superposition on the nucleic acids.

Table S4: Variance of the dynamics explained by the first three eigenvectors

	DNA	dsRNA	GRE_RNA	Gas5_model1	Gas5_model2
Unbound	57.9%	66.4%	65.1%	64.0%	63.5%
GR-bound					
(1)	52.5%	79.0%	58.5%	68.2%	51.1%
(2)	44.7%	65.7%	65.8%	61.3%	56.9%

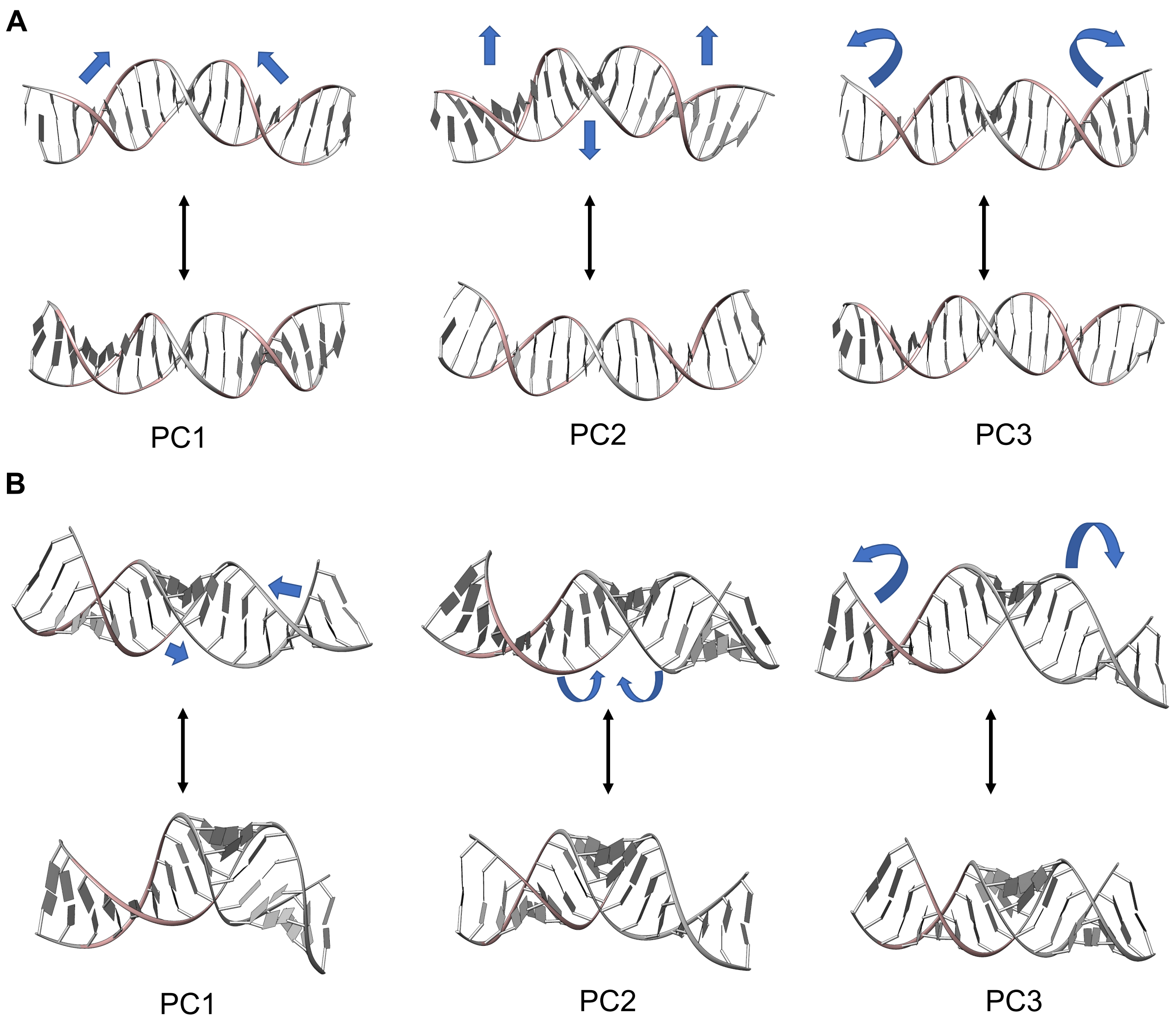

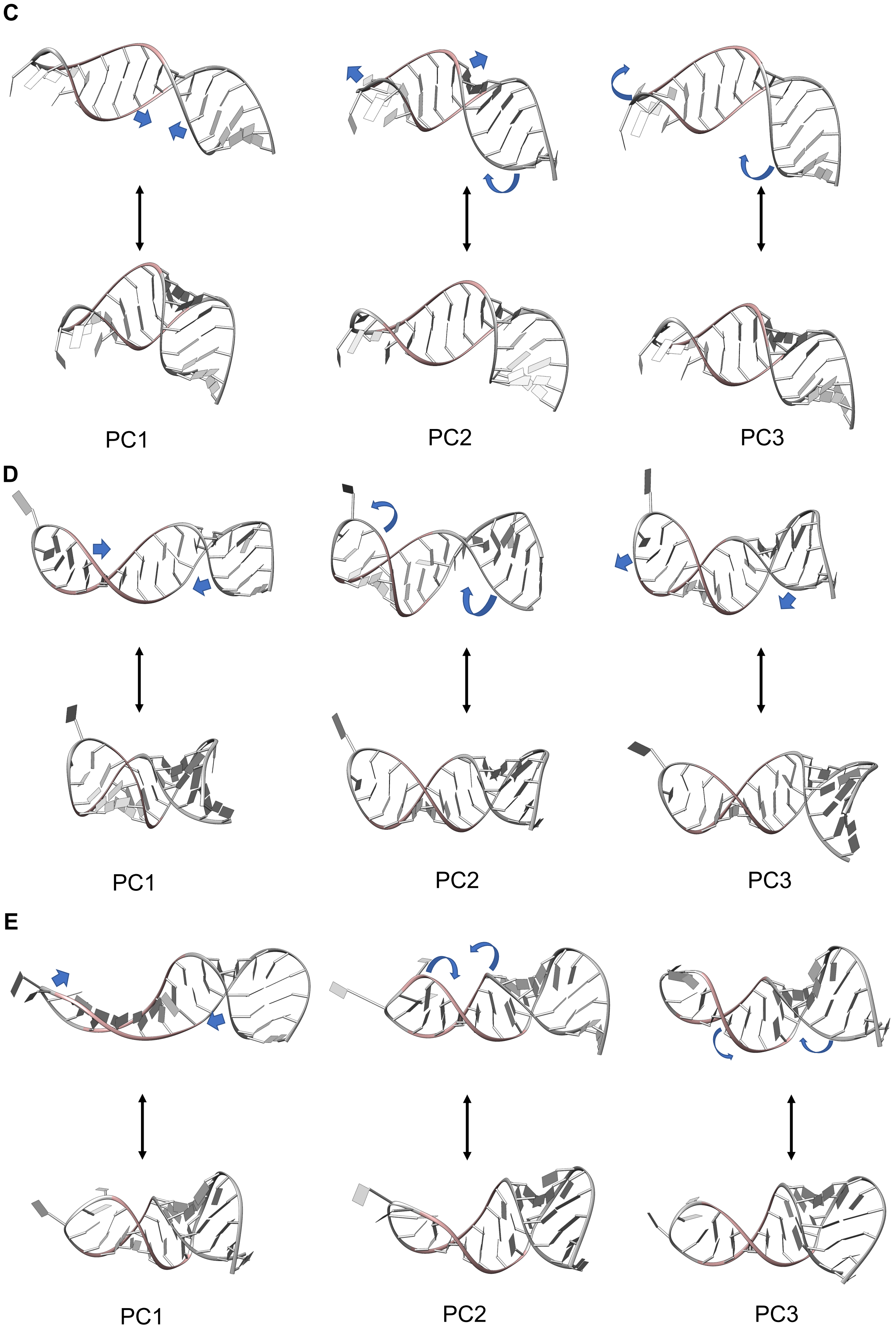
C
PC1
PC2
PC3
D
PC1
PC2
PC3
E
PC1
PC2
PC3

Figure S25: The extreme points for the first three PCs of the unbound nucleic acid systems. **A.** DNA **B.** dsRNA **C.** GRE_RNA **D.** Gas5_RNA_model1 **E.** Gas5_RNA_model2. The blue arrows indicate the motion described by the corresponding PC.

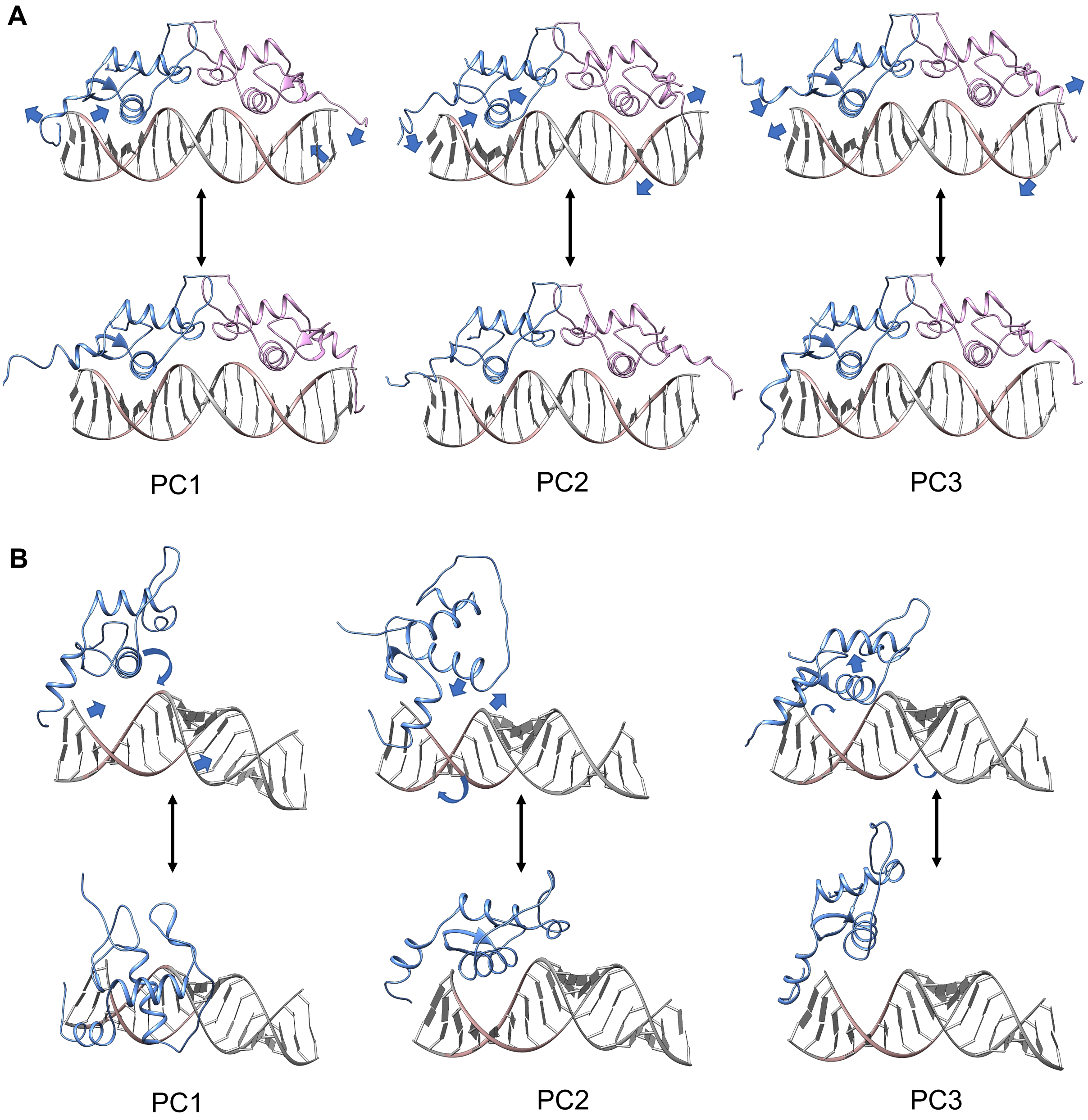

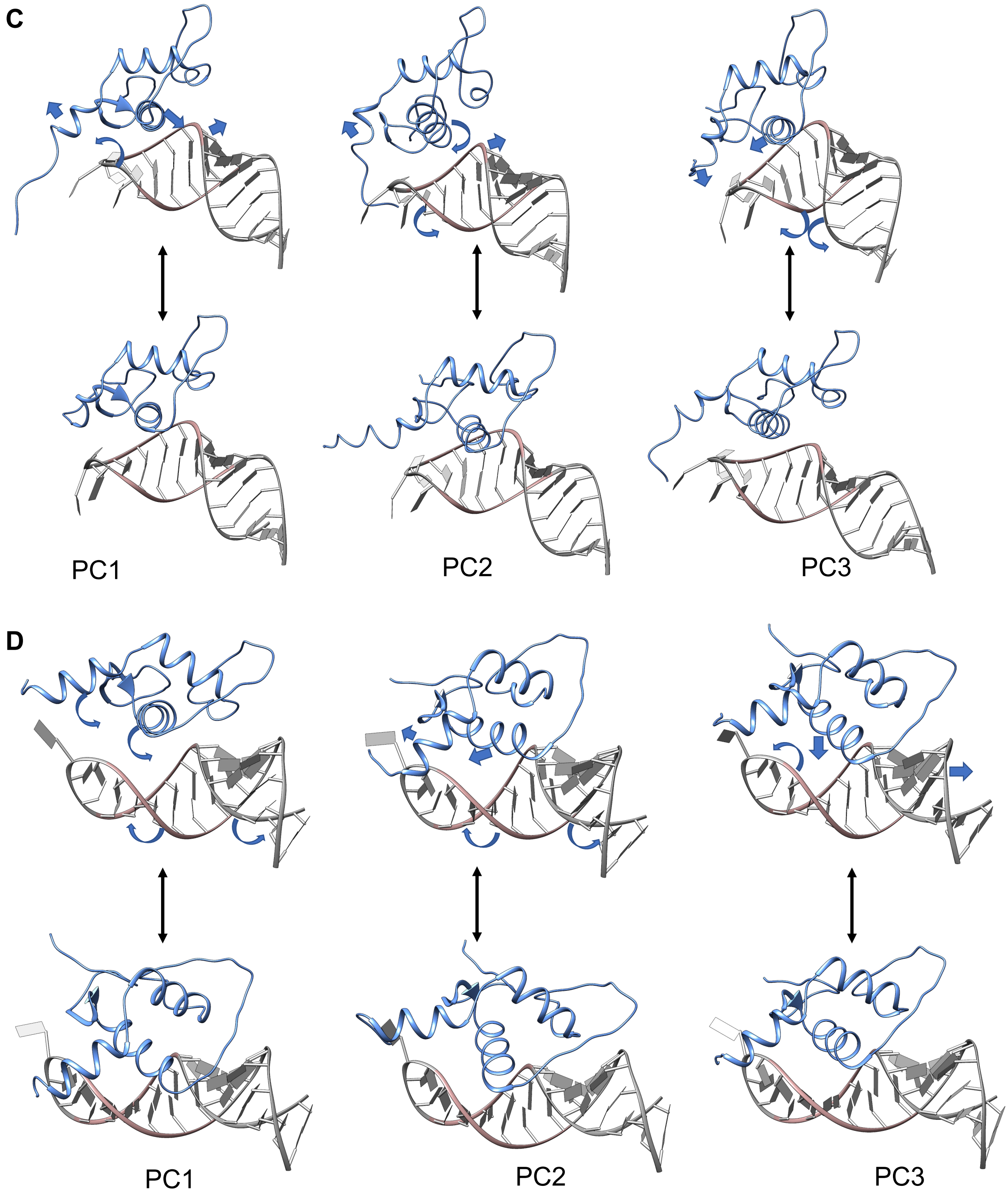
C
PC1
PC2
PC3
D
PC1
PC2
PC3

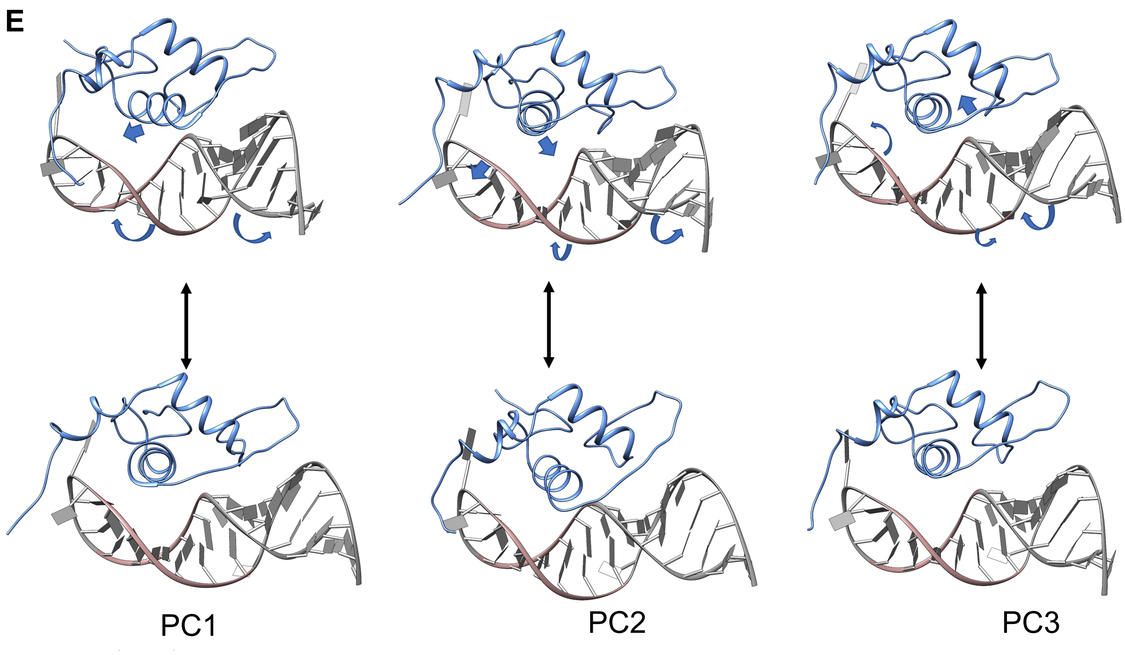

Figure S26: The extreme points for the first three PCs of the GR-bound **A.** DNA **B.** dsRNA **C.** GRE_RNA **D.** Gas5_RNA_model1 **E.** Gas5_RNA_model2. Representative motions for analysis (1). Where analysis (1) is performed for the heavy atoms of the protein-DNA/RNA systems with the superposition on the nucleic acids. The blue arrows indicate the motion described by the corresponding PC.

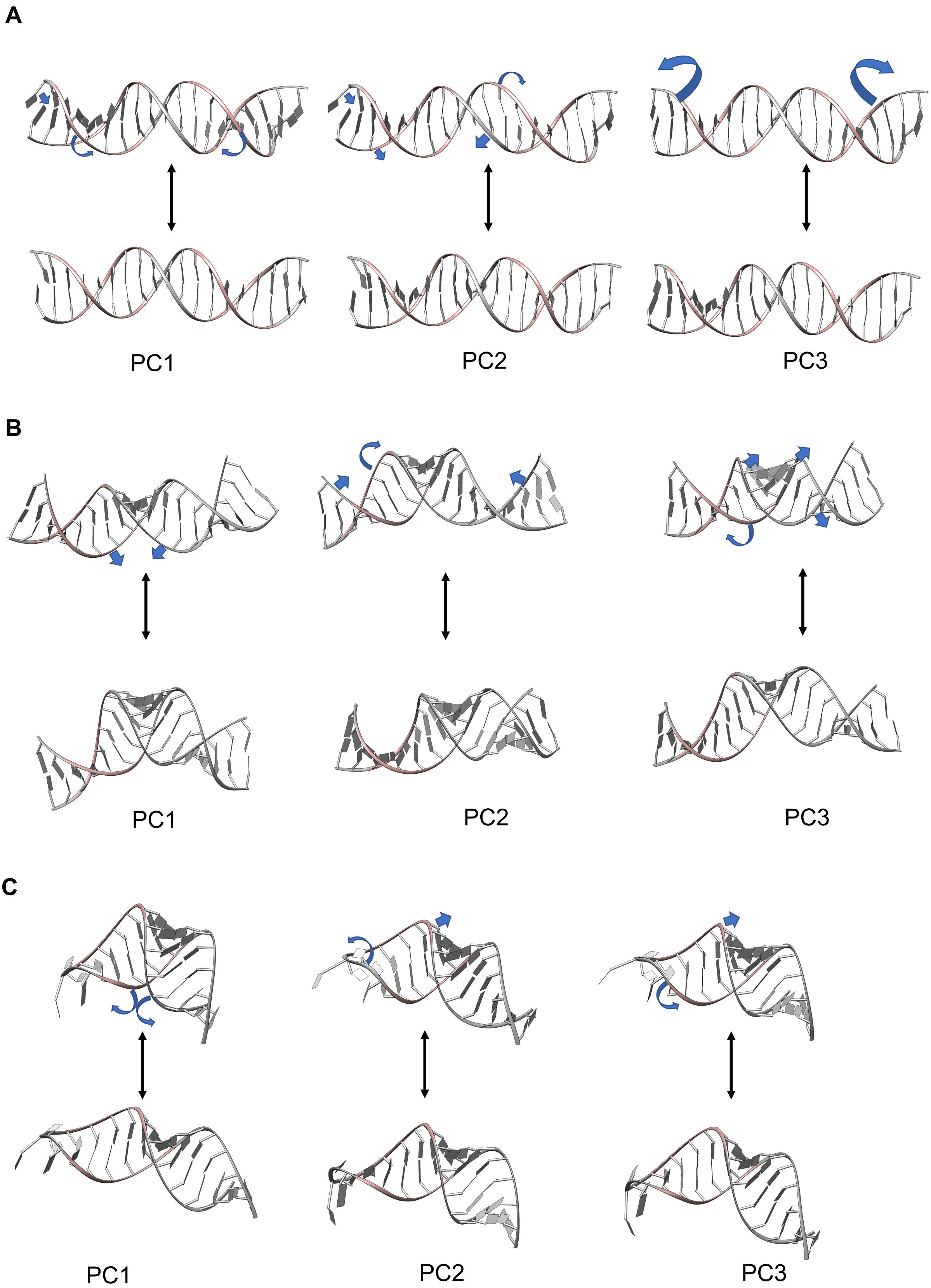

A
PC1
PC2
PC3
B
PC1
PC2
PC3
C
PC1
PC2
PC3

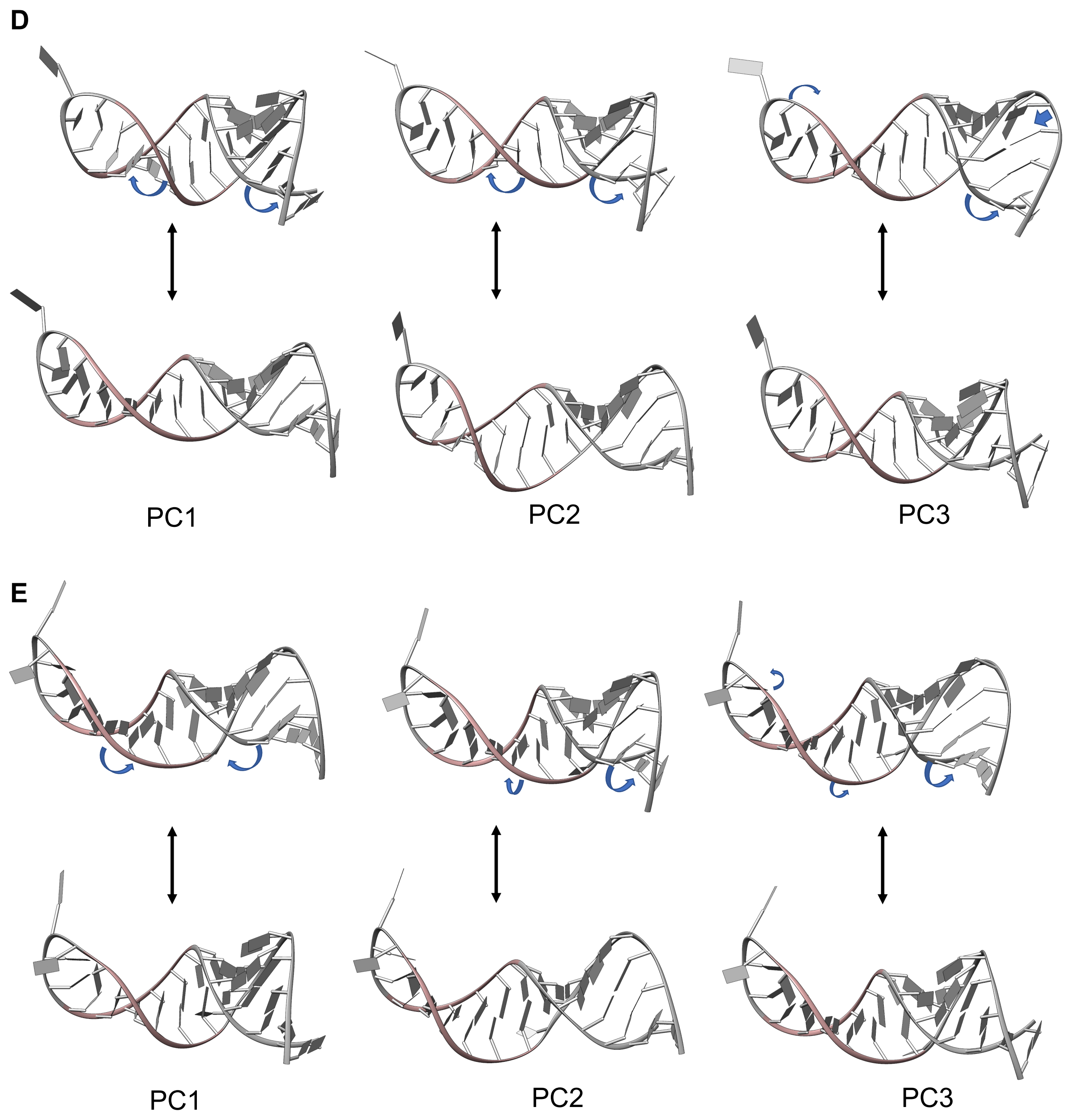

Figure S27: The extreme points for the first three PCs of the GR-bound **A.** DNA **B.** dsRNA **C.** GRE_RNA **D.** Gas5_RNA_model1 **E.** Gas5_RNA_model2. Representative motions for analysis (2), which is performed for heavy atoms of DNA/RNA with the superposition on the nucleic acids. The blue arrows indicate the motion described by the corresponding PC.
